# Pyridinium-2-carbaldoximes with quinolinium carboxamide moiety are simultaneous reactivators of acetylcholinesterase and butyrylcholinesterase inhibited by nerve agent surrogates

**DOI:** 10.1080/14756366.2020.1869954

**Published:** 2021-01-19

**Authors:** Hyun Myung Lee, Rudolf Andrys, Jakub Jonczyk, Kyuneun Kim, Avinash G. Vishakantegowda, David Malinak, Adam Skarka, Monika Schmidt, Michaela Vaskova, Kamil Latka, Marek Bajda, Young-Sik Jung, Barbara Malawska, Kamil Musilek

**Affiliations:** a Division of Bio and Drug Discovery, Korea Research Institute of Chemical Technology, Daejeon, Republic of Korea; b Department of Medicinal Chemistry and Pharmacology, Daejeon, Republic of Korea; c Department of Chemistry, Faculty of Science, University of Hradec Kralove, Hradec Kralove, Czech Republic; d Department of Physicochemical Drug Analysis, Faculty of Pharmacy, Jagiellonian University Medical College, Krakow, Poland

**Keywords:** Organophosphate, acetylcholinesterase, butyrylcholinesterase, reactivator, oxime

## Abstract

The pyridinium-2-carbaldoximes with quinolinium carboxamide moiety were designed and synthesised as cholinesterase reactivators. The prepared compounds showed intermediate-to-high inhibition of both cholinesterases when compared to standard oximes. Their reactivation ability was evaluated *in vitro* on human recombinant acetylcholinesterase (*hr*AChE) and human recombinant butyrylcholinesterase (*hr*BChE) inhibited by nerve agent surrogates (NIMP, NEMP, and NEDPA) or paraoxon. In the reactivation screening, one compound was able to reactivate *hr*AChE inhibited by all used organophosphates and two novel compounds were able to reactivate NIMP/NEMP-*hr*BChE. The reactivation kinetics revealed compound **11** that proved to be excellent reactivator of paraoxon-*hr*AChE better to obidoxime and showed increased reactivation of NIMP/NEMP-*hr*BChE, although worse to obidoxime. The molecular interactions of studied reactivators were further identified by *in silico* calculations. Molecular modelling results revealed the importance of creation of the pre-reactivation complex that could lead to better reactivation of both cholinesterases together with reducing particular interactions for lower intrinsic inhibition by the oxime.

## 1. Introduction

Organophosphates (OPs) in the form of nerve agents (e.g. sarin, VX tabun) or pesticides (e.g. paraoxon as more toxic metabolite of parathion) are serious threat or health problem in a global perspective^1^. They irreversibly inhibit acetylcholinesterase (AChE), butyrylcholinesterase (BChE), or other esterases responsible for crucial processes in the organism^2^. However, the most acute for human health is AChE inhibition. In principle, OP is irreversibly bound to Ser203 (*h*AChE) and causes accumulation of acetylcholine on the synapses. The synaptic overstimulation with consequent nicotinic, muscarinic, or central effects may lead to serious injury or death^3^.

**Figure 1.**
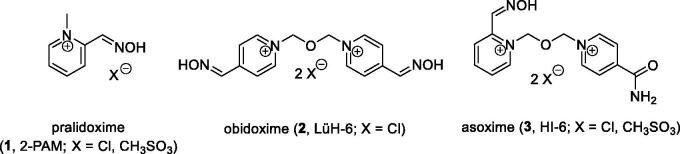
Standard acetylcholinesterase reactivators used as the causal antidotes.

The OP intoxication can be treated by cholinesterase antidotes. They are usually composed of parasympatholytic agent (e.g. atropine and avizafone), cholinesterase reactivator (e.g. pralidoxime **1**, obidoxime **2**, and asoxime **3**; Figure 1), and anticonvulsant (e.g. diazepam)^4^. The symptomatic treatment is mediated by parasympatholytics and anticonvulsants. However, the causal treatment is done by cholinesterase reactivator with oxime moiety. The oxime functional group is able to transform into nucleophilic oximate anion under physiological pH 7.4 and split the OP moiety from AChE active site via formation of phosphylated oxime that is further degraded or excreted^5^. This way, OP is detoxified and AChE function is restored.

The AChE reactivators (so called oximes) have very variable ability to detoxify various OP species^6^. In fact, there is no optimal oxime able to treat all OP intoxications. In clinical praxis, the charged oximes are used (e.g. Figure 1). Their biggest advantage consists in a high water solubility that allows their rapid distribution after i.m. administration and a high clearance from organism via urine^7^. However, their disadvantage is very limited CNS distribution and thus limited possibility to reactivate brain AChE^8^. To overlap this issue, there are recent studies that are focussed on encapsulation of charged reactivators into, e.g. solid lipid nanoparticles that were found to improve reactivation of brain AChE^9^.

For this reason, various uncharged oximes or non-oxime reactivators were introduced and they were recently reviewed^10^. Their advantage should consist in higher CNS permeability and thus more rapid reactivation of brain AChE. Some studies suggested the uncharged oximes are bioavailable in CNS, but the rapid elimination from the CNS was also proved^11^^,^^12^. In contrast, the non-oxime reactivators failed to provide effective OP detoxification to date^13^.

In the last decade, a particular attention was focussed on reactivation of BChE as well. The BChE is natural bioscavenger that is binding OP without serious physiological consequences. On this basis, the pseudo-catalytic scavenger concept was proposed^14^. Such antidote should consist of *h*BChE and its effective reactivator, which would allow to repeatedly restore BChE scavenging function. For this purpose, the purification system for truly human BChE from human plasma was developed^15^. However, there is lack of effective BChE reactivators since the charged or uncharged oximes were designed to primarily reactivate AChE. Recently, the promising and simultaneous AChE and BChE reactivation was found for e.g. charged chlorinated oximes^16^^,^^17^.

**Figure 2.**
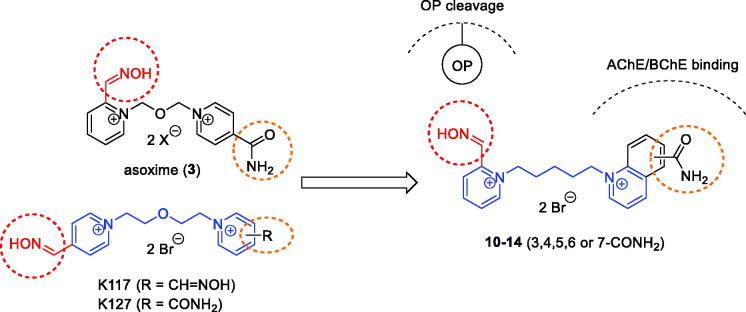
Molecular design of novel pyridinium-2-carbaldoximes with quinolinium carboxamide moiety.

In this work, we were focussed on design, synthesis and detailed *in vitro/in silico* evaluation of charged pyridinium-2-carbaldoximes on human recombinant AChE (*hr*AChE) and human recombinant BChE (*hr*BChE) inhibited by nerve agent surrogates (NIMP, NEMP, and NEDPA) and pesticide metabolite paraoxon. The novel oximes were designed from formerly effective and simultaneous AChE/BChE reactivators K117 or K127 (Figure 2) with changed structural motif for binding in cholinesterase active site.

## 2. Experimental

### 2.1. Chemistry

#### 2.1.1. General information

Unless otherwise stated, all commercially available reagents and solvents were used without further purification. DMF, CH_3_CN, CH_2_Cl_2_, and THF were dried by using a JC Meyer solvent purification system prior to use. Analytical thin layer chromatography (TLC) was performed on Merck silica gel 60 F_254_ plates. Column chromatography was performed on silica gel 60 (230–400 mesh) using proper eluent systems. ^1^H NMR was recorded on Bruker 500 instrument at 500 MHz and ^13^C NMR was recorded at 125 MHz. Chemical shifts were quoted in parts per million (ppm) referenced to the appropriate solvent peak or 0.0 ppm for tetramethylsilane. The following abbreviations were used to describe peak splitting patterns when appropriate: br = broad, s = singlet, d = doublet, t = triplet, q = quartette, dd = doublet of doublet, td = triplet of doublet, ddd = doublet of doublet of doublet, and m = multiplet. Coupling constants, *J*, were reported in hertz unit (Hz). ^13^C NMR was recorded on Bruker Avance instrument at 125 MHz and fully decoupled by broad band proton decoupling. Chemical shifts are given in ppm referenced to the centre of a heptate at 40.01–39.01 ppm of DMSO-d_6_. High resolution mass spectra (HRMS) were obtained by using EI ionisation and ESI ionisation methods. All compounds assayed were >95% pure, as determined by UPLC analysis conducted on Waters Acquity UPLC H-Class system with photodiode array (PDA) detector using a reverse-phase column with a linear H_2_O/CH_3_CN gradient system, 10% to 90% CH_3_CN in H_2_O.

#### 2.1.2. Synthesis of pyridinium-2-carbaldoximes with quinolinium carboxamide moiety

##### 2.1.2.1. Synthesis of carboxamide precursors

*Quinoline-3-carboxamide (****4****) was purchased from Combi-Blocks. Quinoline-4-carboxamide* (**5**): To a mixture of quinoline-4-carboxylic acid (6.00 g, 37 mmol) in CH_2_Cl_2_ (690 mL) was added nine drops of DMF at 0 °C, followed by oxalyl chloride (16 mL) dropwise. The resulting reaction was stirred at room temperature (r.t.) overnight, and then concentrated *in vacuo*. The crude mixture was dissolved in THF (300 mL) and ammonia gas was added to the solution at 0 °C. After 1 h, the reaction mixture was concentrated *in vacuo*, filtered and washed with water to give the desired product (3.96 g, 66%). ^1^H NMR (500 MHz, DMSO-d_6_) *δ* 8.97 (d, *J* = 4.2 Hz, 1H), 8.26 (s, 1H), 8.22 (d, *J* = 8.4 Hz, 1H), 8.08 (d, *J* = 8.4 Hz, 1H), 7.93 (s, 1H), 7.81 (t, *J* = 7.6 Hz, 1H), 7.68 (t, *J* = 7.6 Hz, 1H), 7.57 (d, *J* = 4.2 Hz, 1H).

*Quinoline-5-carboxamide (****6****) was purchased from AA blocks. Quinoline-6-carboxamide* (**7**): To a mixture of quinoline-6-carboxylic acid (12.8 g, 74 mmol) in CH_2_Cl_2_ (740 mL) was added 20 drops of DMF at 0 °C, followed by oxalyl chloride (32 mL) dropwise. The resulting reaction was stirred at r.t. overnight, and then concentrated *in vacuo*. The crude mixture was dissolved in THF (600 mL) and ammonia gas was added to the solution at 0 °C. After 1 h, the reaction mixture was concentrated *in vacuo*, filtered and washed with water to give the desired product (6.0 g, 47%). ^1^H NMR (500 MHz, DMSO-d_6_) *δ* 8.99 (dd, *J* = 4.2, 1.7 Hz, 1H), 8.58 (d, *J* = 1.5 Hz, 1H), 8.52–8.43 (m, 1H), 8.29 (s, 1H), 8.22 (dd, *J* = 8.8, 1.9 Hz, 1H), 8.07 (d, *J* = 8.8 Hz, 1H), 7.62 (dd, *J* = 8.3, 4.2 Hz, 2H).

*Quinoline-7-carboxamide* (**8**): To a mixture of quinoline-7-carboxylic acid (0.50 mg, 2.9 mmol) in CH_2_Cl_2_ (29 mL, 0.1 M) was added one drops of DMF at 0 °C, followed by oxalyl chloride (0.4 mL, 5 mmol) dropwise. The resulting reaction was stirred at r.t. for 1 h, and then concentrated *in vacuo*. The crude mixture was dissolved in NH_4_OH (50 mL) and stirred at r.t. for 0.5 h. The reaction mixture was concentrated *in vacuo*, added with NaHCO_3_ and extracted with EA. The combined organic layers were washed with brine, dried over Na_2_SO_4_, filtered and concentrated *in vacuo* to give the desired product (371 mg, 74%). ^1^H NMR (500 MHz, DMSO-d_6_) *δ* 8.99 (dd, *J* = 4.2, 1.7 Hz, 1H), 8.58 (s, 1H), 8.43 (dd, *J* = 8.3, 0.9 Hz, 1H), 8.29 (s, 1H), 8.05 (d, *J* = 1.0 Hz, 2H), 7.62 (dd, *J* = 8.3, 4.2 Hz, 2H).

##### 2.1.2.2. Synthesis of monoquaternary precursor

*1-(5-Bromopentyl)-2-((hydroxyimino)methyl)pyridin-1-ium bromide (****9****)*. 1,5-Dibromopentane (84 mL, 615 mmol) was dissolved in MeCN (373 mL, 0.55 M) and added 2-pyridinealdoxime (25 g, 205 mmol). The reaction mixture was stirred at 85 °C for 75 h. The reaction mixture was cooled to r.t. and removed the solvent. The crude mixture was dissolved in MeOH and silica gel (10 g) was added to the crude mixture. The solution was stirred at r.t. for 30 min and concentrated *in vacuo*. The solid was packing with silica gel and purified by silica gel column chromatography. After further purification by silica gel column chromatography and recrystallisation in acetone/MeOH to give the desired product (7.46 g, 13.4%). ^1^H NMR (500 MHz, DMSO-d_6_) *δ* 13.14 (s, 1H), 9.10 (d, *J* = 6.0 Hz, 1H), 8.89–8.71 (m, 1H), 8.56 (t, *J* = 7.9 Hz, 1H), 8.41 (dd, *J* = 8.2 Hz, 1H), 8.12 (t, *J* = 6.2 Hz, 1H), 4.89–4.66 (m, 2H), 3.53–3.52 (m, 2H), 2.00–1.65 (m, 4H), 1.60–1.37 (m, 2H). ^13^C NMR (125 MHz, DMSO-d_6_) *δ* 146.77, 146.00, 145.62, 141.46, 127.48, 125.93, 57.57, 34.81, 31.45, 29.40, and 24.02. HRMS calculated for C_11_H_16_BrN_2_O^+^ 271.0441 [M–H]^+^; found: *m/z* 271.0451.

##### 2.1.2.3. Synthesis of final bisquaternary salts

*3-Carbamoyl-1-(5-(2-((hydroxyimino)methyl)pyridin-1-ium-1-yl)pentyl)quinolin-1-ium bromide (****10****)*. 1-(5-Bromopentyl)-2-((hydroxyimino)methyl)pyridin-1-ium bromide (736 mg, 2.09 mmol) and quinoline-3-carboxamide (300 mg, 1.74 mmol) were dissolved in DMF (17 mL). The reaction mixture was stirred at 85 °C for 144 h. Acetone was poured into the reaction mixture and keep in refrigerator overnight. The solid was filtered and washed with acetone to give the desired product (170 mg, 17%). ^1^H NMR (500 MHz, DMSO-d_6_) *δ* 13.14 (s, 1H), 10.07 (s, 1H), 9.80 (s, 1H), 9.14 (d, *J* = 6.2 Hz, 1H), 8.79 (s, 1H), 8.75–8.65 (m, 2H), 8.57 (q, *J* = 8.1 Hz, 2H), 8.45–8.32 (m, 2H), 8.22 (s, 1H), 8.13 (t, *J* = 7.4 Hz, 2H), 5.15 (t, *J* = 7.4 Hz, 2H), 4.78 (t, *J* = 7.7 Hz, 2H), 2.26–1.97 (m, 2H), 1.99–1.72 (m, 2H), and 1.68–1.35 (m, 2H). ^13^C NMR (125 MHz, DMSO-d_6_) *δ* 163.14, 149.20, 146.75, 145.98, 145.81, 145.21, 141.44, 141.37, 137.85, 136.97, 131.74, 130.46, 128.76, 127.67, 127.45, 125.87, 57.51, 57.29, 29.64, 28.80, and 22.33. HRMS calculated for C_21_H_24_N_4_O_2_^2+^ 363.1888 [M–H]^+^; found: *m/z* 363.1881.

*4-Carbamoyl-1-(5-(2-((hydroxyimino)methyl)pyridin-1-ium-1-yl)pentyl)quinolin-1-ium bromide (****11****)*. 1-(5-Bromopentyl)-2-((hydroxyimino)methyl)pyridin-1-ium bromide (736 mg, 2.09 mmol) and quinoline-4-carboxamide (300 mg, 1.74 mmol) were dissolved in DMF (17 mL). The reaction mixture was stirred at 85 °C for 144 h. Acetone was poured into the reaction mixture and keep in refrigerator overnight. The solid was filtered and washed with acetone to give the desired product (65 mg, 7%). ^1^H NMR (500 MHz, DMSO-d_6_) *δ* 13.14 (s, 1H), 10.07 (s, 1H), 9.80 (s, 1H), 9.14 (d, *J* = 6.2 Hz, 1H), 8.79 (s, 1H), 8.75–8.65 (m, 2H), 8.57 (q, *J* = 8.1 Hz, 2H), 8.45–8.32 (m, 2H), 8.22 (s, 1H), 8.13 (t, *J* = 7.4 Hz, 2H), 5.15 (t, *J* = 7.4 Hz, 2H), 4.78 (t, *J* = 7.7 Hz, 2H), 2.26–1.97 (m, 2H), 1.99–1.72 (m, 2H), and 1.68–1.35 (m, 2H). ^13^C NMR (125 MHz, DMSO-d_6_) *δ* 165.89, 151.76, 149.98, 146.91, 145.91, 145.21, 141.36, 137.88, 135.71, 130.61, 127.93, 127.46, 126.04, 125.80, 119.63, 119.41, 57.51, 57.30, 29.64, 28.85, and 22.34. HRMS calculated for C_21_H_24_N_4_O_2_^2+^ 363.1888 [M–H]^+^; found: *m/z* 363.1880.

*5-Carbamoyl-1-(5-(2-((hydroxyimino)methyl)pyridin-1-ium-1-yl)pentyl)quinolin-1-ium bromide (****12****)*. 1-(5-Bromopentyl)-2-((hydroxyimino)methyl)pyridin-1-ium bromide (736 mg, 2.09 mmol) and quinoline-4-carboxamide (300 mg, 1.74 mmol) were dissolved in DMF (17 mL). The reaction mixture was stirred at 85 °C for 144 h. Acetone was poured into the reaction mixture and keep in refrigerator overnight, then filtered. The solid was put in MeCN at 85 °C for overnight to get the desired product (147 mg, 13%). ^1^H NMR (500 MHz, DMSO-d_6_) *δ* 13.20 (s, 1H), 9.64 (dd, *J* = 5.9, 1.4 Hz, 1H), 9.55–9.48 (m, 1H), 9.07 (dd, *J* = 6.3, 1.4 Hz, 1H), 8.76 (s, 1H), 8.72 (d, *J* = 9.1 Hz, 1H), 8.56 (td, *J* = 7.9, 1.4 Hz, 1H), 8.47 (s, 1H), 8.40 (dd, *J* = 8.2, 1.6 Hz, 1H), 8.32–8.25 (m, 1H), 8.22 (dd, *J* = 8.8, 5.8 Hz, 1H), 8.18 (d, *J* = 7.1 Hz, 1H), 8.13–8.01 (m, 2H), 5.10 (t, *J* = 7.5 Hz, 2H), 4.74 (t, *J* = 7.6 Hz, 2H), 2.12–1.97 (m, 2H), 1.93–1.85 (m, 2H), and 1.55–1.42 (m, 2H). ^13^C NMR (125 MHz, DMSO-d_6_) *δ* 168.30, 150.44, 147.23, 146.53, 145.74, 145.50, 141.92, 138.08, 136.62, 135.12, 129.34, 127.97, 127.44, 126.41, 123.26, 121.47, 57.99, 30.13, 29.33, and 22.77. HRMS calculated for C_21_H_24_N_4_O_2_^2+^ 363.1888 [M–H]^+^; found: *m/z* 363.1879.

*6-Carbamoyl-1-(5-(2-((hydroxyimino)methyl)pyridin-1-ium-1-yl)pentyl)quinolin-1-ium bromide (****13****)*. 1-(5-Bromopentyl)-2-((hydroxyimino)methyl)pyridin-1-ium bromide (500 mg, 1.42 mmol) and quinoline-6-carboxamide (196 mg, 1.14 mmol) were dissolved in DMF (14 mL). The reaction mixture was stirred at 85 °C for 144 h. Acetone was poured into the reaction mixture and keep in refrigerator overnight. The solid was filtered and washed with acetone to give the desired product (40 mg, 6.7%). ^1^H NMR (500 MHz, DMSO-d_6_) *δ* 13.16 (s, 1H), 9.68 (d, *J* = 5.6 Hz, 1H), 9.37 (d, *J* = 8.3 Hz, 1H), 9.09 (d, *J* = 6.1 Hz, 1H), 9.00 (s, 1H), 8.78 (s, 1H), 8.72 (d, *J* = 9.3 Hz, 1H), 8.63 (d, *J* = 9.2 Hz, 1H), 8.57 (t, *J* = 7.8 Hz, 1H), 8.51 (s, 1H), 8.42 (d, *J* = 7.7 Hz, 1H), 8.32–8.23 (m, 1H), 8.12 (t, *J* = 6.6 Hz, 1H), 7.91 (s, 1H), 5.10 (t, *J* = 7.2 Hz, 2H), 4.75 (t, *J* = 7.4 Hz, 2H), 2.16–1.98 (m, 2H), 1.97–1.81 (m, 2H), and 1.61–1.35 (m, 2H). ^13^C NMR (125 MHz, DMSO-d_6_) *δ* 165.75, 150.77, 148.30, 146.77, 145.20, 141.38, 138.45, 134.82, 133.52, 130.36, 129.32, 127.45, 125.84, 122.84, 119.42, 57.49, 57.06, 29.64, 28.82, and 22.27. HRMS calculated for C_21_H_24_N_4_O_2_^2+^Br^–^_2_ 521.0188 [M–H]^–^; found: *m/z* 521.0189. HRMS calculated for C_21_H_24_N_4_O_2_^2+^ 363.1888 [M–H]^+^; found: *m/z* 363.1886.

*7-Carbamoyl-1-(5-(2-((hydroxyimino)methyl)pyridin-1-ium-1-yl)pentyl)quinolin-1-ium bromide (****14****)*. 1-(5-Bromopentyl)-2-((hydroxyimino)methyl)pyridin-1-ium bromide (736 mg, 2.09 mmol) and quinoline-7-carboxamide (300 mg, 1.74 mmol) were dissolved in DMF (17 mL). The reaction mixture was stirred at 85 °C for 144 h. Acetone was poured into the reaction mixture and kept in refrigerator overnight. The solid was filtered and washed with acetone to give the desired product (143 mg, 16%). ^1^H NMR (500 MHz, DMSO-d_6_) *δ* 13.19 (s, 1H), 9.65 (dd, *J* = 5.9, 1.5 Hz, 1H), 9.34 (d, *J* = 8.3 Hz, 1H), 9.05 (dd, *J* = 6.3, 1.4 Hz, 1H), 8.93–8.83 (m, 1H), 8.77 (s, 1H), 8.67–8.62 (m, 1H), 8.60–8.46 (m, 2H), 8.42 (td, *J* = 8.4, 1.4 Hz, 2H), 8.27 (dd, *J* = 8.4, 5.7 Hz, 1H), 8.10 (ddd, *J* = 7.8, 6.2, 1.6 Hz, 1H), 8.04 (s, 1H), 5.16 (t, *J* = 7.6 Hz, 2H), 4.73 (t, *J* = 7.6 Hz, 2H), 2.18–1.99 (m, 2H), 1.92–1.87 (m, 2H), and 1.59–1.39 (m, 2H). ^13^C NMR (125 MHz, DMSO-d_6_) *δ* 165.90, 150.80, 147.11, 146.76, 145.99, 145.22, 141.38, 139.75, 137.16, 131.07, 130.85, 128.26, 127.45, 125.85, 123.33, 117.73, 57.46, 57.06, 29.62, 28.85, and 22.30. HRMS calculated for C_21_H_24_N_4_O_2_^2+^Br^–^_2_ 521.0188 [M–H]^–^; found: *m/z* 521.0187. HRMS calculated for C_21_H_24_N_4_O_2_^2+^ 363.1888 [M–H]^+^; found: *m/z* 363.1885.

### 2.2. *In vitro* assays

#### 2.2.1. Preparation and purification of cholinesterases

The *hr*AChE and *hr*BChE were prepared as recombinant proteins at the University of Hradec Kralove. For their production, the mammalian expression system was used. Briefly, the DNA sequence encoding human AChE and BChE was obtained from UniProtKB Server (www.uniprot.org, accession numbers: P22303 and P06276) and *de novo* synthesised as GeneArt Strings DNA fragments by GeneArt Gene Synthesis Service (Thermo Fisher Scientific, Waltham, MA). The DNA fragments were PCR-amplified using gene-specific primers, adding the DNA sequence for C-terminal 6 × His-tag. The amplicons were inserted into the mammalian pcDNA3.4 vector by TOPO cloning technology, and the final DNA constructs were verified by Sanger sequencing (ABI PRISM 3130xl). For protein expression, the DNA constructs were transiently transfected into Hek293 derivatives. Recombinant proteins were collected from culture supernatant six days later and stored at −80 °C for further purification.

The *hr*AChE and *hr*BChE were purified using NGC Medium-Pressure Chromatography System (Bio-Rad, Hercules, CA)^18^. The total volume of 6–8 mL of medium containing secreted protein was desalted using 5 mL HiTrap Desalting column (GE Healthcare, Chicago, IL) equilibrated with buffer A (20 mM sodium phosphate buffer, 150 mM NaCl, 15 mM imidazole, and 20% glycerol; pH 7.4). Acquired supernatant was loaded onto a 1 mL HisTrap FF column (GE Healthcare, Chicago, IL) equilibrated with buffer A. The captured proteins were eluted with buffer B (20 mM sodium phosphate buffer, 150 mM NaCl, 500 mM imidazole, and 20% glycerol; pH 7.4). Imidazole was subsequently removed by repeated centrifugation in Amicon Ultra-4 (Ultracel-10K) tube (Merck Millipore, Billerica, MA). Protein concentration was determined by linearised Bradford method adapted for 96-well plate.

#### 2.2.2. Inhibition of cholinesterases

The catalytic activity of purified enzymes was determined by standard Ellman method adapted for 96-well plates^19^. The reaction mixture contained purified (uninhibited) *hr*AChE (70 ng/mL protein final concentration) or *hr*BChE (220 ng/mL protein), tested oxime in Na-phosphate buffer at required concentration (varying from 0.1 µM up to 80 µM) and 500 µM 5,5′-dithiobis-2-nitrobenzoic acid (DTNB) in 20 mM Na-phosphate buffer (pH 7.4). This mixture was pre-incubated at 37 °C for 15 min. Further, substrate acetylthiocholine iodide (ATChI) or butyrylthiocholine iodide (BTChI) were added when their final concentrations in the mixture were 1000 µM (the final volume was 100 µL). The catalytic activity was evaluated for further 10 min at 37 °C as amount of product (5-thio-2-nitrobenzoic acid, TNB) formed by enzyme spectrophotometrically at 436 nm using Spark multimode microplate reader (Tecan, Männedorf, Switzerland). The intrinsic inhibitory activity of individual oximes (represented by IC_50_ values) towards *hr*AChE or *hr*BChE was determined by non-linear regression using GraphPad Prism 8.2 (GraphPad Software, La Jolla, CA).

#### 2.2.3. Reactivation screening

4-Nitrophenyl isopropyl methylphosphonate (NIMP, sarin surrogate; >95%), 4-nitrophenyl ethyl methylphosphonate (NEMP, VX surrogate, >95%), and 4-nitrophenyl ethyl dimethylphosphoramidate (NEDPA, tabun surrogate, >95%) were purchased commercially from Chemforase (Mont-Saint-Aignan, France). The ethylparaoxon (POX, >99%) was purchased from Merck (Prague, Czech Republic). The purified *hr*AChE or *hr*BChE was inhibited by 25 µM NIMP, NEMP, NEDPA, or POX for 30 min in order to obtain >99% inhibition. The excess of OP was removed by dialysis against 25 mM Na-phosphate buffer (pH 7.4) for 16 h (three buffer exchanges) at 4–6 °C. The enzymatic activity of purified (uninhibited) *hr*AChE as a control or inhibited *hr*AChE was evaluated prior or post dialysis to ensure that it had minimal influence towards uninhibited or inhibited enzyme (Figure S1; Supplementary information). The OP-inhibited enzyme was incubated for 15 min with 100 or 10 µM solution of tested oxime. The reaction mixture consisted of 10 µL of inhibited enzyme (1.95 ng of total protein), 20 µL of 2.5 mM DTNB, 10 µL of corresponding oxime solution, and 50 µL of 25 mM Na-phosphate buffer (pH 7.4). The reaction was started by addition of 10 µL of 10 mM substrate ATChI or BTChI (final volume was 100 µL). The catalytic activity of reactivated enzyme was measured spectrophotometrically at 436 nm using Spark multimode microplate reader (Tecan, Männedorf, Switzerland).

#### 2.2.4. Reactivation kinetics

Selected compounds with promising reactivation ability were further analysed in order to obtain reactivation kinetics parameters. Inhibited enzyme was incubated in eight different times (varying from 0.5 to 60 min) with seven different concentrations of tested oxime (varying from 5 to 800 µM) spectrophotometrically at 436 nm using Spark multimode microplate reader (Tecan, Männedorf, Switzerland). Acquired data were analysed by non-linear regression analysis according to Worek et al.^20^ using GraphPad Prism 8.2 (GraphPad Software, La Jolla, CA).

### 2.3. Computational methods

The three-dimensional ligand structures were built with the Corina online tool (Molecular Networks GmbH, Erlangen, Germany & Altamira). Oximes were prepared in the anionic form. Before docking, atom types and protonation states of ligand structures were checked and Gasteiger-Marsili charges were assigned using Sybyl-X 1.1 (Tripos, St. Louis, MO). All proteins used in the docking studies were prepared with ProteinPrepare web service (pH 7.4, without water molecules, including heteroatoms in p*K*_a_ calculation)^21^. Docking studies were divided into three parts. In the first part, the inhibition of AChE and BChE by oximes was performed on mouse apo enzymes, i.e. AChE (PDB code: 1J06) and human BChE (PDB code: 1P0I) with the Gold Suite 5.3 (The Cambridge Crystallographic Data Centre, Cambridge, UK). The binding site was defined with all amino acid residues within 15 Å from the serine of the catalytic triad. A standard set of genetic algorithm parameters with a population size of 100 and a number of operations of 100,000 was applied. As a result, 10 ligand conformations were obtained and sorted according to the values of scoring function – GoldScore.

The second part, the interactions of oximes during the creation of non-reactivating complexes with AChE and BChE inhibited by OPs (VX, sarin, paraoxon) were performed with AChE complexes (PDB codes: 2Y2V, 2Y2U, and 5HF5) and human BChE (PDB code: 3DJY) with oximes transferred from AChE complexes. Docking was conducted with the Gold v5.3 program using analogical docking parameters to those applied in the first part of the studies. In the third stage, previously used protein complexes were modified and the conformations of the OPs in them correspond to the sarin conformation observed in the 5FPP complex. Docking studies were performed with the SkeleDock web service to map the pre-reactivation position of the pyridinium oxime fragment observed in the 5FPP complex with asoxime^22^. Final poses were optimised and rescored with the Gold v5.3 program (The Cambridge Crystallographic Data Centre, Cambridge, UK). During rescoring, the binding site was defined with all amino acid residues within 15 Å from the serine of the catalytic triad. Rescoring was performed with the GoldScore scoring function. All results were visualised by PyMOL 0.99rc2 (DeLano Scientific LLC, Palo Alto, CA).

## 3. Results and discussion

### 3.1. Molecular design and synthesis

Pyridinium-2-carbaldoximes (e.g. pralidoxime **1**, asoxime **3**) and pyridinium-4-carbaldoximes (e.g. obidoxime **2**) are known to effectively reactivate OP-inhibited AChE. Their reactivation properties are given by 2- or 4-positioned oxime moiety, which has p*K*_a_ ∼7.4–8.0 and it allows efficient formation of oximate anion at pH 7.4 in human organism^23^. However, their reactivation of OP-inhibited BChE was found limited^24^. Their analogues with 4-positioned oxime and pyridinium-4-carboxamide moiety (e.g. K027, K048, K203, and K868) were described to have similar effectiveness for certain OP-inhibited AChE and valuable carboxamide binding in the AChE peripheral site^16^^,^^25^. However again, their reactivation of OP-inhibited BChE was rather limited with exception of K868^17^. But a decade ago, there were promising charged oximes found for AChE or BChE reactivation (K117 and K127, Figure 2) that were highlighted for tabun inhibited-BChE and recently showed slightly increased brain penetration in mice^14^^,^^26^^,^^27^.

In this study, the molecular fragments of promising AChE and BChE reactivators were incorporated in one molecule (Figure 2). First, oxime moiety was retained in position 2 on the pyridinium scaffold to decrease p*K*_a_ for effective reactivation similarly to **1** or **3**. Second, the quinolinium amide moiety was used for binding into PAS of AChE similarly to K-oximes or for binding within more spacious BChE active site^28^. In addition, the linker consisting of five methylene units was implemented as a fragment for valuable AChE or BChE reactivation similarly to K117 and K127, but optimally with a weaker inhibition of both enzymes^28–30^.

**Scheme 1.**
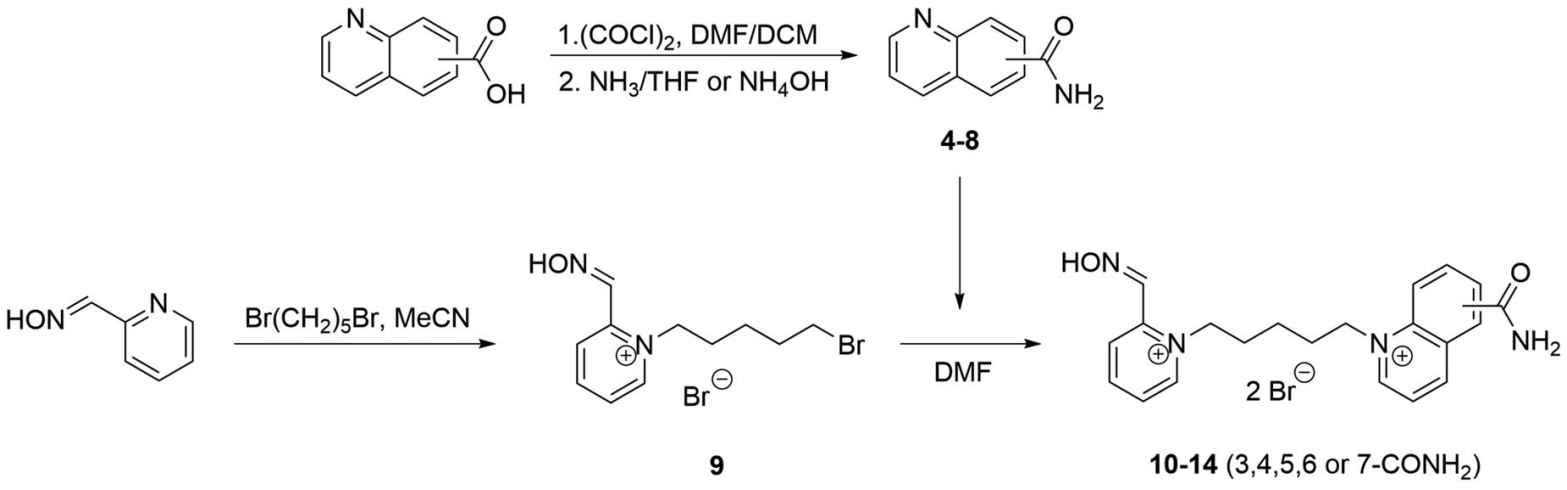
Synthesis of novel pyridinium-2-carbaldoximes with quinolinium carboxamide moiety.

The pyridinium-2-carbaldoximes with quinolinium carboxamide moiety (**4**–**8**) were prepared in three step synthesis (Scheme 1). First, the corresponding quinolinium carboxamide was synthesised from its carboxylic acid using *in situ* acyl halide formation. Second, pyridine-2-carbaldoxime was treated with dibromopentane to form monocharged salt in acetonitrile (MeCN). Finally, the products were prepared by attaching the quinolinium carboxamides in DMF and characterised.

### 3.2. *In vitro* inhibition and reactivation

The prepared compounds and standards were first tested on *hr*AChE and *hr*BChE inhibition (Tables 1 and 2) to determine their intrinsic inhibitory ability. The *hr*AChE in the solution was used for *in vitro* screening purposes as a model of human AChE that is mostly membrane bound in the neuronal tissues. The *hr*BChE in the solution was used as model of human BChE from plasma. The standard oximes were found weak inhibitors of both enzymes. In contrast, the novel compounds resulted as low µM inhibitors of *hr*AChE with exception of oxime **11** (IC_50_ ∼18 µM). Plus, they showed low to high inhibition of *hr*BChE with oximes **11** and **13** (IC_50_ ∼45 µM) being the less potent BChE inhibitors. The lower BChE inhibition is valuable to retain BChE scavenging function to bind OP while OP-BChE adduct can be further reactivated. As such, BChE in combination with BChE reactivator can detoxify multiple OP molecules.

**Table 1.** Reactivation screening of *hr*AChE inhibited by nerve agent surrogates and paraoxon.

Compound	PAS ligand	*hr*AChE	*hr*AChE reactivation (%)							
		IC_50_±CI_95%_ (µM)	NIMP ± SD		NEMP ± SD		NEDPA ± SD		POX ± SD	
			100 µM	10 µM	100 µM	10 µM	100 µM	10 µM	100 µM	10 µM
pralidoxime (**1**)	–	629 ± 35.9	44.4 ± 0.6	10.7 ± 0.4	28.3 ± 0.9	4.5 ± 0.4	26.4 ± 0.1	4.3 ± 0.5	36.6 ± 0.5	5.6 ± 0.1
obidoxime (**2**)	Pyr-4-CH=NOH	330 ± 38.0	72.5 ± 3.3	54.4 ± 3.7	57.7 ± 2.1	11.5 ± 1.2	49.5 ± 4.7	31.7 ± 0.9	94.9 ± 1.6	34.5 ± 1.6
asoxime (**3**)	Pyr-4-CONH_2_	145 ± 11.8	63.3 ± 3.2	43.7 ± 1.3	88.2 ± 6.8	24.2 ± 1.2	22.6 ± 1.8	2.6 ± 0.2	21.8 ± 2.8	2.3 ± 0.3
KR-26351 (**10**)	Quin-3-CONH_2_	2.31 ± 0.5	6.1 ± 0.1	7.3 ± 0.77	5.0 ± 0.2	4.3 ± 0.2	5.9 ± 0.3	9.0 ± 0.7	7.3 ± 0.7	12.4 ± 1.0
KR-26352 (**11**)	Quin-4-CONH_2_	18.3 ± 1.6	15.8 ± 0.2	14.3 ± 1.0	10.0 ± 0.6	6.2 ± 0.1	13.9 ± 1.0	21.4 ± 1.2	21.0 ± 2.74	23.2 ± 1.1
KR-26353 (**12**)	Quin-5-CONH_2_	1.52 ± 0.3	4.5 ± 0.1	3.6 ± 0.3	1.7 ± 0.2	1.7 ± 0.6	2.4 ± 0.2	4.0 ± 0.2	5.6 ± 1.0	6.9 ± 0.9
KR-26354 (**13**)	Quin-6-CONH_2_	1.08 ± 0.3	8.0 ± 0.7	9.9 ± 1.2	5.3 ± 0.4	8.2 ± 0.8	4.7 ± 0.2	9.3 ± 1.1	4.7 ± 0.7	9.8 ± 1.3
KR-26355 (**14**)	Quin-7-CONH_2_	0.37 ± 0.2	1.6 ± 0.1	1.4 ± 0.1	1.4 ± 0.1	0.7 ± 0.6	1.7 ± 0.4	1.5 ± 0.5	3.0 ± 1.1	2.3 ± 0.5

**Table 2.** Reactivation screening of *hr*BChE inhibited by nerve agent surrogates and paraoxon.

			*hr*BChE reactivation (%)							
		*hr*BChE	NIMP ± SD		NEMP ± SD		NEDPA ± SD		POX ± SD	
Compound	PAS ligand	IC_50_±CI_95%_ (µM)	100 µM	10 µM	100 µM	10 µM	100 µM	10 µM	100 µM	10 µM
pralidoxime (**1**)	–	>3500	55.8 ± 1.4	15.2 ± 0.7	29.5 ± 0.4	6.5 ± 0.3	9.6 ± 0.7	1.30 ± 0.1	2.9 ± 0.7	0.4 ± 0.2
obidoxime (**2**)	Pyr-4-CH=NOH	760 ± 41.0	53.2 ± 3.5	27.1 ± 0.9	25.1 ± 0.4	9.1 ± 0.2	12.1 ± 0.4	3.50 ± 0.6	4.9 ± 0.2	1.2 ± 0.2
asoxime (**3**)	Pyr-4-CONH_2_	1363 ± 260.0	45.9 ± 0.5	14.0 ± 0.8	20.7 ± 0.5	3.6 ± 0.2	3.8 ± 0.5	1.00 ± 0.6	3.8 ± 0.7	0.7 ± 0.4
KR-26351 (**10**)	Quin-3-CONH_2_	15.03 ± 2.4	13.6 ± 2.2	12.0 ± 1.1	9.5 ± 0.2	8.4 ± 0.7	6.3 ± 0.9	2.8 ± 1.0	5.0 ± 0.04	1.0 ± 0.3
KR-26352 (**11**)	Quin-4-CONH_2_	44.93 ± 3.6	33.3 ± 0.6	24.7 ± 0.5	26.5 ± 2.5	23.4 ± 1.2	9.5 ± 0.1	–	7.5 ± 1.0	–
KR-26353 (**12**)	Quin-5-CONH_2_	6.58 ± 2.0	2.8 ± 0.6	3.7 ± 0.3	5.8 ± 0.9	5.0 ± 0.2	–	–	1.0 ± 0.6	–
KR-26354 (**13**)	Quin-6-CONH_2_	44.73 ± 4.3	23.9 ± 0.1	22.3 ± 0.4	22.2 ± 0.5	5.9 ± 0.2	0.5 ± 0.1	–	–	–
KR-26355 (**14**)	Quin-7-CONH_2_	0.61 ± 0.2	11.8 ± 0.6	3.5 ± 0.2	3.5 ± 0.9	0.8 ± 0.4	–	–	4.4 ± 1.0	–

The reactivation screening was done on *hr*AChE and *hr*BChE inhibited by nerve agent surrogates (NIMP – sarin surrogate, NEMP – VX surrogate, NEDPA – tabun surrogate) and pesticide metabolite paraoxon (POX). The nerve agent surrogates were used for safety reasons, when they produce the same cholinesterase adducts as the real nerve agents, but they have decreased toxicity^31^. The dialysis method was used to remove the excess of OP agent from the OP-inhibited enzyme. The conditions of used dialysis protocol (4–6 °C, pH 7.4) ensured the minimal ageing of OP moiety bound to active site of the enzyme^32^. For reactivation of *hr*AChE, obidoxime (**2**) was found to be the best reactivator for NIMP, NEDPA, and POX, while asoxime (**3**) resulted as the lead compound in case of NEMP inhibition. The best reactivation among novel compounds was found for oxime **11**, which was able to restore *hr*AChE inhibited by all tested OPs, while the other novel compounds were found ineffective. The higher reactivation presented by compound **11** is most probably related to its lower AChE inhibition when compared to other tested oximes.

For reactivation of *hr*BChE, pralidoxime (**1**), obidoxime (**2**), and asoxime (**3**) resulted very closely in reactivation of NIMP or NEMP at higher concentration (100 µM). However, only obidoxime (**2**) showed particular reactivation of NIMP-*hr*BChE and negligible reactivation of NEMP-*hr*BChE in one order of magnitude lower concentration (10 µM). Further, two novel compounds **11** and **13** were found to effectively reactivate NIMP- and NEMP-*hr*BChE. Interestingly, the reactivation of both novel oximes was comparable with obidoxime for NIMP-*hr*BChE at 10 µM, and compound **13** resulted as best oxime for reactivation of NEMP-*hr*BChE at 10 µM. Again, both compounds resulted as weaker BChE inhibitors and better reactivators when compared to the other novel oximes in the series. There was found minimal reactivation of NEDPA- or POX-i*hr*BChE by all tested oximes.

**Figure 3.**
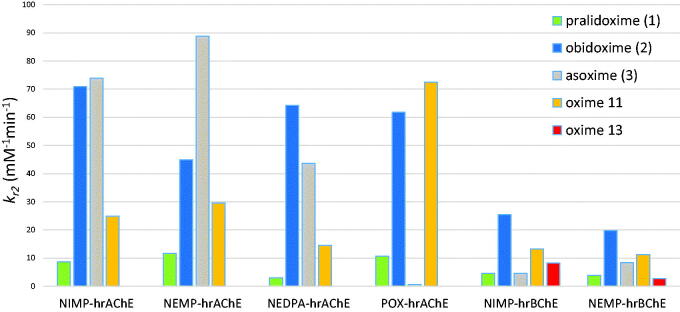
Comparison of overall reactivation rate constants (*k*_r2_) of inhibited *hr*AChE and *hr*BChE.

Based on reactivation screening, the reactivation kinetics was further determined for all standards (**1**–**3**), compound **11** in case of *hr*AChE and compounds **11** and **13** in case of *hr*BChE (Tables 3 and 4, Figure 3). The affinity of oximes towards OP-inhibited enzymes (reflected by *K*_D_) and their ability to remove the phosphyl residue from the active site of the enzyme (reflected by the reactivity constant *k*_r_) were determined, when the specific overall second-order reactivation rate constants (*k*_r2_=*k*_r_/*K*_D_) were calculated.

**Table 3.** Reactivation kinetics of *hr*AChE inhibited by nerve agent surrogates and paraoxon by selected oximes.

	NIMP-*hr*AChE			NEMP-*hr*AChE			NEDPA-*hr*AChE			POX-*hr*AChE		
Compound	*K*_D_ (µM)	*k*_r_ (min^–1^)	*k*_r2_ (mM^–1^ min^–1^)	*K*_D_ (µM)	*k*_r_ (min^–1^)	*k*_r2_ (mM^–1^ min^–1^)	*K*_D_ (µM)	*k*_r_ (min^–1^)	*k*_r2_ (mM^–1^ min^–1^)	*K*_D_ (µM)	*k*_r_ (min^–1^)	*k*_r2_ (mM^–1^ min^–1^)
pralidoxime (**1**)	102.7	0.889	8.66	87.22	1.024	11.74	51.89	0.151	2.92	97.04	1.039	10.71
obidoxime (**2**)	36.05	2.554	70.85	54.68	2.456	44.92	33.98	2.181	64.18	30.50	1.883	61.74
asoxime (**3**)	9.274	0.685	73.89	15.28	1.356	88.74	12.32	0.537	43.60	164.50	0.104	0.63
KR-26352 (**11**)	8.654	0.216	24.95	10.56	0.313	29.59	33.48	0.486	14.50	7.86	0.567	72.48

*K*_D_: phosphylated enzyme-oxime dissociation constant; *k*_r_: first-order reactivation rate constant; *k*_r2_: second-order reactivation rate constant (*k*_r2_=*k*_r_/*K*_D_).

**Table 4.** Reactivation kinetics of *hr*BChE inhibited by NIMP, NEMP, and paraoxon by selected oximes.

Compound	NIMP-*hr*BChE			NEMP-*hr*BChE		
	*K*_D_ (µM)	*k*_r_ (min^–1^)	*k*_r2_ (mM^–1^ min^–1^)	*K*_D_ (µM)	*k*_r_ (min^–1^)	*k*_r2_ (mM^–1^ min^–1^)
pralidoxime (**1**)	415.60	1.891	4.55	827.2	3.257	3.94
obidoxime (**2**)	81.29	2.065	25.40	147	2.898	19.71
asoxime (**3**)	227.80	1.052	4.62	176.1	1.475	8.38
KR-26352 (**11**)	12.81	0.170	13.27	15.59	0.175	11.21
KR-26354 (**13**)	5.31	0.044	8.29	28.11	0.078	2.76

*K*_D_: phosphylated enzyme-oxime dissociation constant; *k*_r_: first-order reactivation rate constant; *k*_r2_: second-order reactivation rate constant (*k*_r2_=*k*_r_/*K*_D_).

In case of NIMP- and NEMP-*hr*AChE, asoxime (**3**) resulted with the highest *k*_r2_ followed by obidoxime (**2**). Compound **11** was found better to pralidoxime (**1**) for NIMP- and NEMP-*hr*AChE in contrast to reactivation screening of NEMP-*hr*AChE at 100 µM. For NEDPA-*hr*AChE, obidoxime (**2**) showed highest *k*_r2_ followed by asoxime (**3**) and oxime **11**, which resulted again better to pralidoxime (**1**). Notably, oxime **11** was found to have the highest overall reactivation constant for POX-*hr*AChE followed by obidoxime (**2**), which is in contrast to its reactivation screening results at both concentrations.

In case of NIMP- and NEMP-*hr*BChE, obidoxime (**2**) showed the best *k*_r2_ values for both inhibitors, whereas pralidoxime (**1**) and asoxime (**3**) resulted with markedly lower overall reactivation rate constants. Interestingly, oximes **11** and **13** *k*_r2_ values were found better to pralidoxime (**1**) and asoxime (**3**) for NIMP-*hr*BChE, plus oxime **11** resulted better for NEMP-*hr*BChE, but in all cases slightly worse to obidoxime (**2**).

To sum up the *in vitro* reactivation data for the novel compounds, oxime **11** showed some reactivation of *hr*AChE inhibited by all tested OP surrogates and POX, when in exhibited the highest overall reactivation rate for POX-*hr*AChE among all tested reactivators. Oximes **11** and **13** showed some reactivation of *hr*BChE inhibited by NIMP or NEMP, when oxime **11** resulted with the best overall reactivation rate, although worse to obidoxime (**2**). Based on these data, the molecular modelling study was performed to reveal interactions of novel oximes with intact or inhibited AChE or BChE with particular attention to oximes **11** and **13**.

### 3.3. Molecular modelling

#### 3.3.1. Acetylcholinesterase and butyrylcholinesterase inhibition

**Figure 4.**
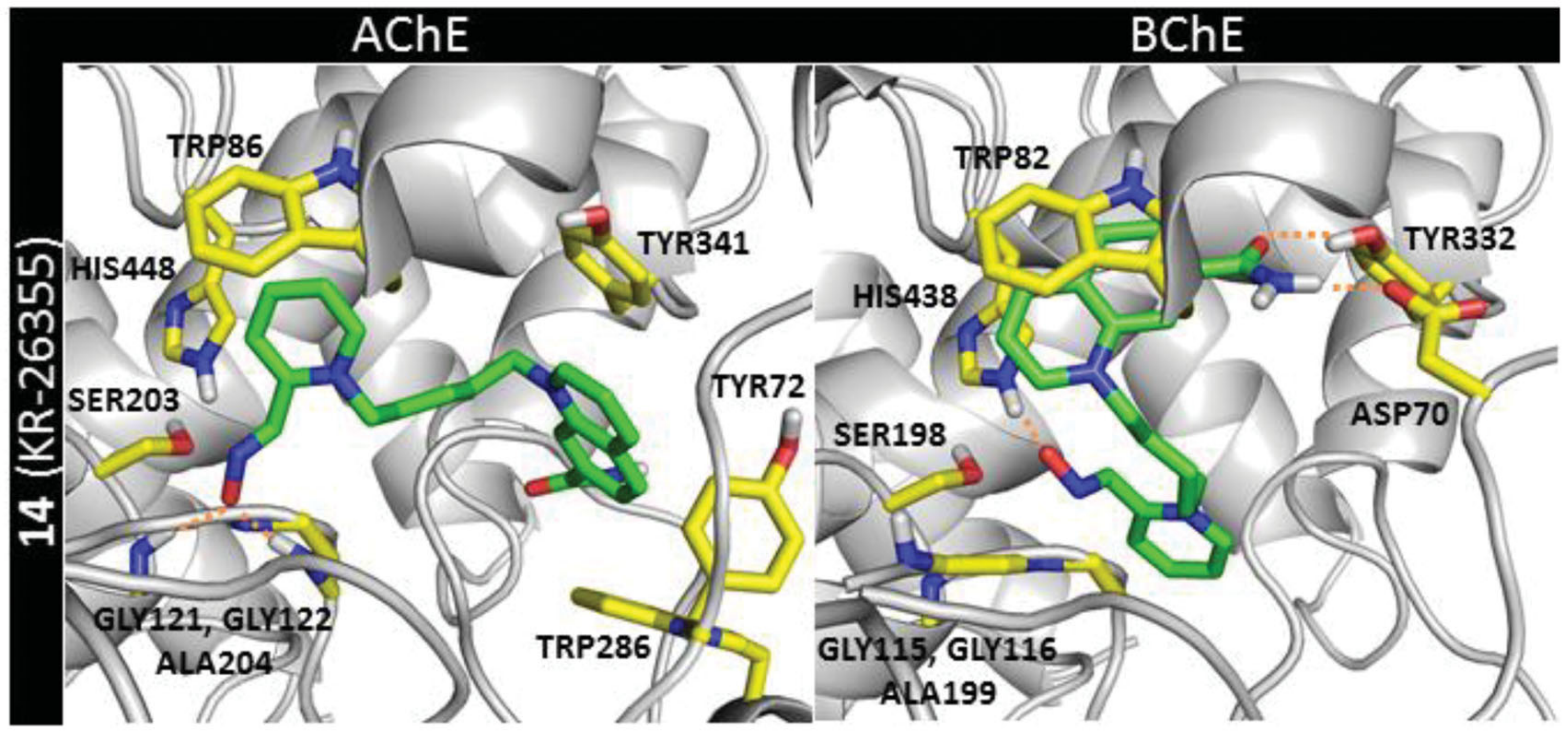
Binding mode of the **14** (marked in green) within AChE and BChE active site.

In molecular modelling experiments, all tested compounds (**10**–**14**) proved to be stronger cholinesterase inhibitors than used standards (**1**–**3**) with the most powerful inhibitor **14** (Figure 4) and the weakest inhibitor **11** for both enzymes. The inhibition potency was inversely correlated with the ability to reactivate enzymes blocked by OPs. In the case of AChE, more potent inhibitors (**12**–**14**) showed very consistent binding mode in the docking studies. The crucial interactions for compound binding were formed by oxime moiety within the oxyanion hole (GLY121, GLY122, and ALA204) as well as cation–π interactions created by the pyridinium moiety with TRP86 and π–π stacking between quinolinium fragment and TYR341. The differences in the position of the amide moiety affect the compound interaction with the peripheral anionic site (PAS). Substitution in position 6 (**13**) and 7 (**14**) promoted the creation of hydrogen bonds with SER293, PHE295, and ARG296. When the amide group was in position 3 (**10**), hydrogen bonds were formed instead of a cation–π interaction with TYR341. And the amide group in position 5 (**12**) did not form hydrogen bonds that could enhance interaction with the enzyme. The substitution in position 4 (**11**) also led to the lack of hydrogen bond formation by the amide group. Moreover, this moiety caused steric hindrance which weakened the interaction of the quinolinium system with TRP341. In contrast the AChE, the entrance to the BChE active site is more spacious which is related to less aromatic amino acids in this region in the case of BChE^33^. This fact causes the differences both in the inhibition ability and in the binding mode of the tested compounds within cholinesterases. For all tested compounds, it was observed that the oxime fragment was bound close to the oxyanion hole of the BChE (GLY115, GLY116, and ALA199) where it created several hydrogen bonds. Moreover, the quinolinium fragment of compounds created cation–π and π–π interactions with TRP82. Similarly to the AChE, the position of the amide group modulated the inhibitory ability of the compounds. For the most potent inhibitor **14**, amide moiety created hydrogen bonds with two amino acids TYR332 and ASP70. The weakest inhibitor **11** still presented interactions with the oxyanion hole but there was found only π–π stacking between quinoline and TRP82 with one additional hydrogen bond created by carboxamide oxygen atom and GLY115.

#### 3.3.2. Reactivation cycle analysis

**Figure 5.**
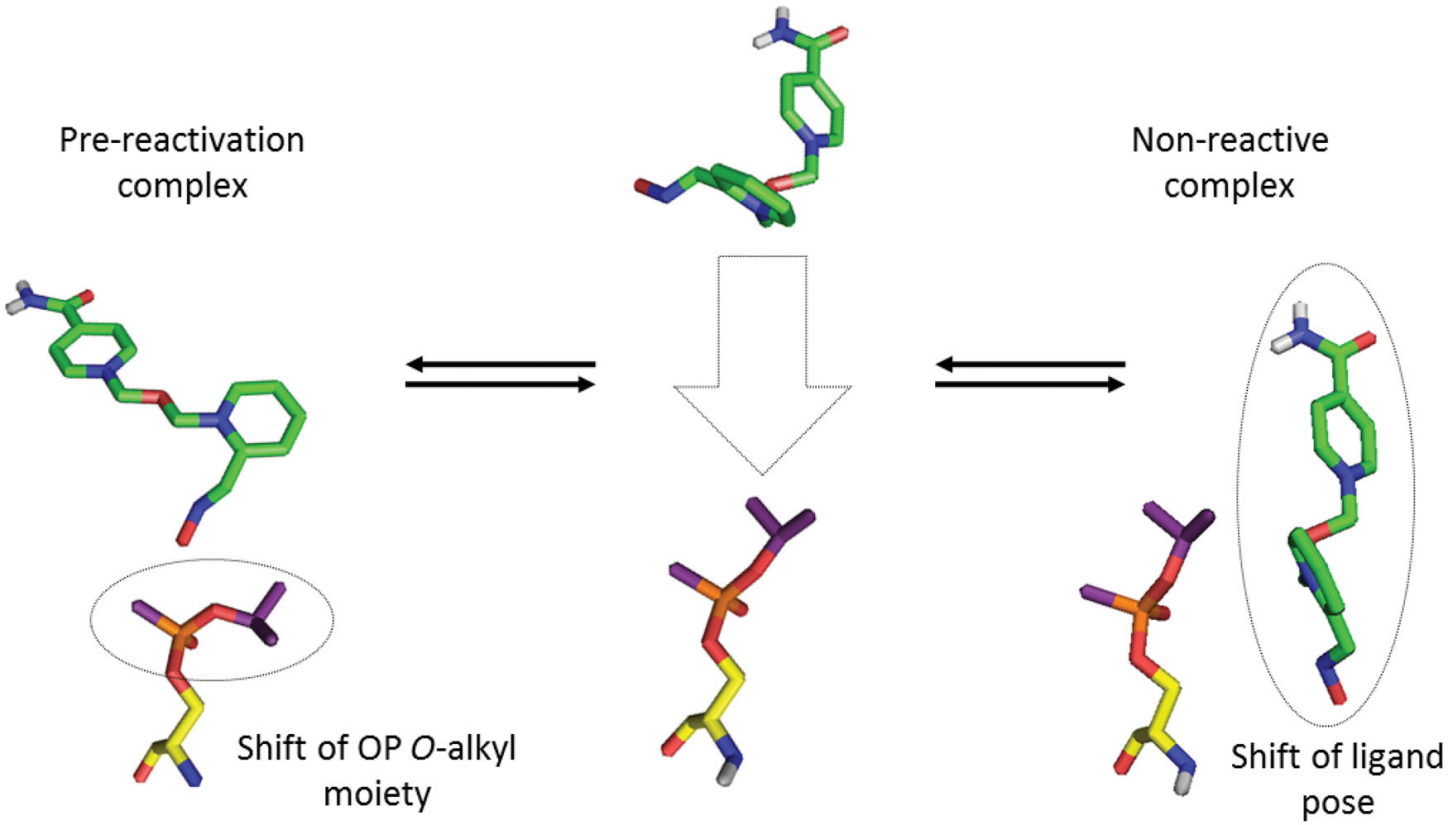
The balance between the pre-reactivation complex (left) and non-reactive complex (right) as exemplified by the interaction of asoxime with sarin-inhibited cholinesterase.

In the theoretical studies on the cholinesterase reactivation, it is crucial to understand, how the oxime compound can interact with the catalytic site of ChE inhibited by OP at the molecular level. In the reactivation cycle proposed by Allgardsson et al.^34^, the system can evolve in two ways after the creation of an initial complex. The first path leads to the formation of a pre-reactivation complex, a next reactive conformation, and as a consequence, to the reactivated enzyme. The creation of a pre-reactivation complex was captured in the crystal structure of *Mus musculus* AChE (*m*AChE)–sarin complex with asoxime (PDB code: 5FPP). The second path leads to the creation of a non-reactive conformation which prevents the compound from performing a nucleophilic attack on OP adduct. Figure 5 shows the relation between these two complexes. For our analysis, we assumed the existence of a balance between the formation of the pre-reactivation and non-reactive complex. We assumed that the equilibrium state between these conformations reflects the effectiveness of reactivating oxime. Analysing the results of the virtual docking via reflecting both conformations, we wanted to indicate potential molecular aspects that might determine the increased or decreased reactivation ability of the individual compounds.

The non-reactive conformations of oxime reactivators can be found in many crystal structures (PDB codes: e.g. 2WHP, 5HFA, and 5FPP). Similar poses were also observed during our docking studies with both novel and standard oximes to the OP-AChE and OP-BChE adducts.

**Figure 6.**
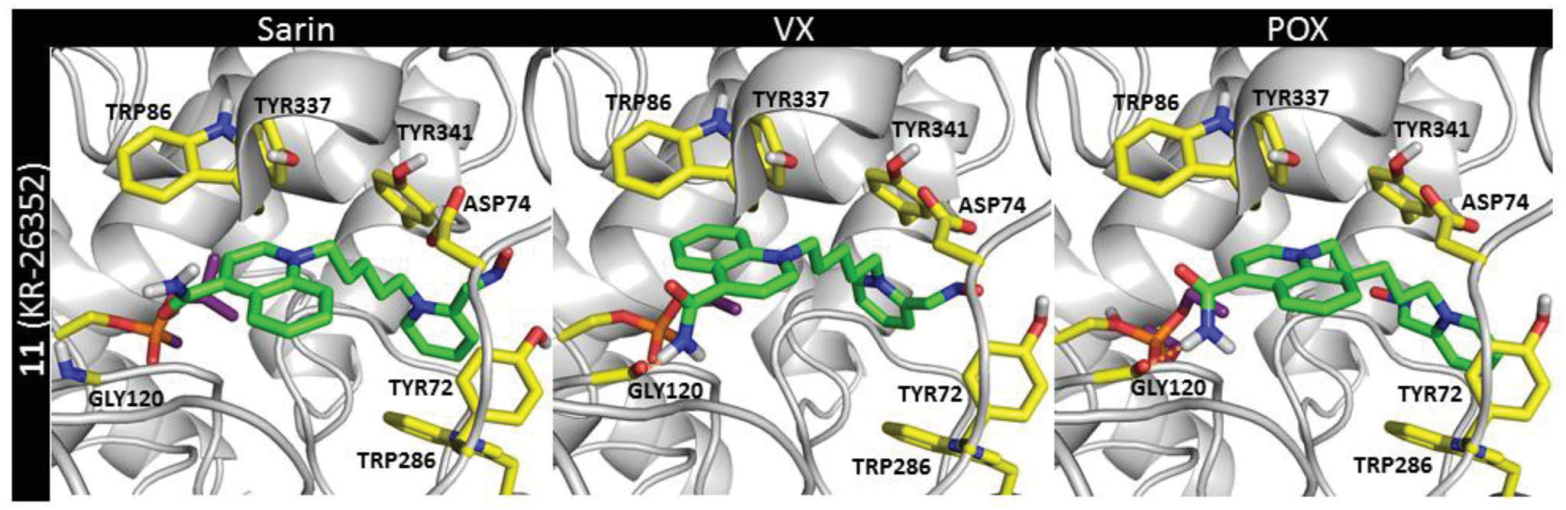
The non-reactive poses of compound **11** obtained during docking to AChE inhibited by sarin (NIMP), VX (NEMP), and POX complexes.

The non-reactive binding modes of obidoxime (**2**) and asoxime (**3**) from docking to AChE were analysed and we observed a significant similarity in their docking results. The recurring π–π stacking and cation–π interactions between the pyridine ring connected to oxime moiety and aromatic TRP86 residue seemed to be crucial for obidoxime and asoxime binding within AChE active site. The arrangement of these standard oximes was additionally stabilised by hydrogen bonds between the oxime moiety and GLY120. The main difference between compounds **2** and **3** was found in their interactions with amino acids within the entrance to the enzyme. In the case of obidoxime, regardless of which OP compound was bound to AChE, the second pyridinium fragment interacted with TYR337 (cation–π interactions) and ASP74 (ionic interactions). In the case of asoxime in the AChE-VX complex, we observed a hydrogen bond between the amide group and the main chain of ASP74. Differently, compound **11** in a non-reactive arrangement preferred to interact via the quinolinium system with TRP86 (Figure 6). However and in diversely from the standard compounds **2**–**3**, these interactions were found weaker and limited to the cation–π interactions. Pyridine ring connected with the oxime moiety of **11** was found to be located within the entrance to the enzyme active site, interacting with TRP286, TYR72, and slightly less often with ASP74 or TYR337. All described poses are presented in Figure 2(S) (Supplementary information).

The next step of our analysis was focussed on the oxime conformation in the pre-reactivation state referring to that observed in the crystal structure (PDB code: 5FPP)^34^. Using the position of pyridinium moiety with oxime of asoxime as a template we reproduced the pre-reactivation position of the tested reactivators. It allowed us to check which of the novel compounds was the best suited to the protein when the reactivation process began. The results of the standard compounds **2** and **3** brought many interesting observations. Most importantly, the poses of individual oximes in the pre-reactivation complexes with sarin-, VX-, and POX-inhibited AChE indicated the existence of repetitive interactions with particular amino acids (Figure 3(S)). The asoxime (**3**) exhibited the largest number of interactions in a complex with sarin-inhibited AChE. These were π–π stacking and cation–π interactions with TYR341, the CH-π interaction with TYR124 and π–π stacking with TRP286. A slight shift of asoxime position was observed in the complex with VX-AChE and it led to weaker interaction with TYR124. In the POX-AChE adduct, such shifted position was even more apparent and it prevented the formation of aromatic interactions with TYR124 and TRP286. The lack of interactions with particular amino acids may explain the observed decline in asoxime reactivation ability for POX-inhibited *hr*AChE (Table 1). In contrast to asoxime, the number of specific interactions for obidoxime (**2**) was only slightly changed. The previously mentioned interactions occurred with the additional ionic interaction with ASP74 presented in all OP-AChE adducts. In the case of POX-AChE, the cation–π interaction with TYR72 was observed. These findings were consistent with the results of *in vitro* experiments in which obidoxime (**2**) maintained the high reactivation ability for all tested OP-AChE adducts.

**Figure 7.**
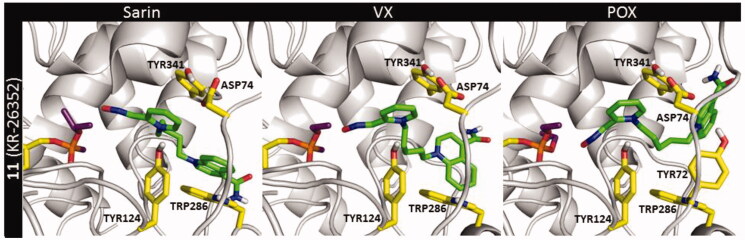
The pre-reactivation poses of compound **11** in AChE blocked by sarin (NIMP), VX (NEMP), and POX.

The most active from the prepared compounds (oxime **11**, Figure 7) showed the highest number of interactions with the AChE inhibited by sarin and POX. During the docking into the VX-AChE adduct, specific interactions were limited to π–π stacking and cation–π interactions with TYR341 and CH-π with TRP286. Such decrease in the amount of specific interactions could be the reason for the observed decline in the reactivation ability of **11** against VX (Table 1).

We conducted an analogous analysis for compounds reactivating BChE blocked by sarin and VX, and compared them to the obidoxime (**2**). The BChE active site is larger than that of AChE. Differences are most visible within the PAS. However and despite the enzymatic differences, the binding mode of obidoxime within the BChE active site was closely similar to that observed with AChE. The π–π stacking and cation–π interactions with TRP82 and TYR332 were observed in the most poses obtained during oxime docking of OP-BChE complexes. All described interactions are presented in Figure 4(S).

**Figure 8.**
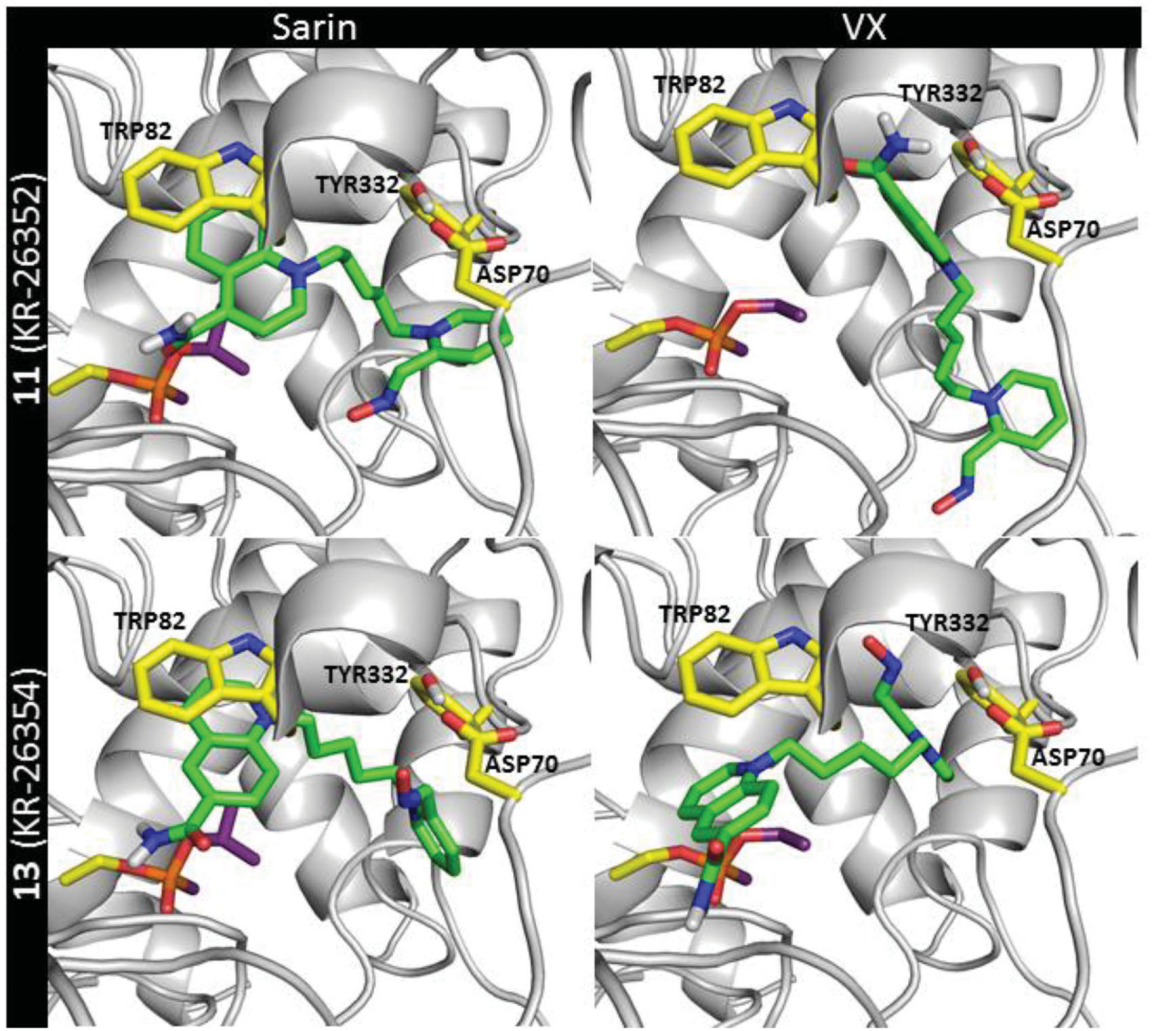
The non-reactive binding mode of compounds **11** and **13** at the BChE active site blocked by sarin (NIMP) and VX (NEMP).

The binding mode in the non-reactive complex for reactivators **11** and **13** with BChE did not change significantly when compared to AChE even though PAS of BChE does not contain aromatic residues corresponding to TRP286 and TYR72 from AChE (Figure 8). The π–π stacking and cation–π interactions with TRP82 and TYR332 were still dominant. Moreover, ASP70 was involved in ionic interactions with both compounds. The only exception was the result obtained for **11** and the VX-BChE adduct when none of the 10 docking runs established a pose corresponding to that for VX-AChE. The highest-scored pose shown in Figure 8 indicated that the compound **11** was bound at the entrance to the active site of the enzyme. In this case, the quinolinium moiety interacted with TYR332 by π–π stacking and cation–π interactions. Further, the quinolinium moiety created an ionic bond with ASP70. The oxime-containing fragment only formed a single hydrogen bond with the ASN289 backbone.

In the next step, we have obtained pre-reactivation poses of OP-BChE adducts via an analogous way to that for AChE. First, the binding mode of obidoxime (**2**) was evaluated. The π–π stacking with TYR332 and ionic bond with ASP70 were the common interactions observed for sarin and VX-inhibited BChE. For sarin-BChE adduct, the additional hydrogen bonds between the second (neutral) oxime group of obidoxime and LEU273 and ALA277 at the entry to the enzyme cavity were formed. Docking results of obidoxime with the VX-BChE adduct did not show these additional interactions (Figure 5(S)).

**Figure 9.**
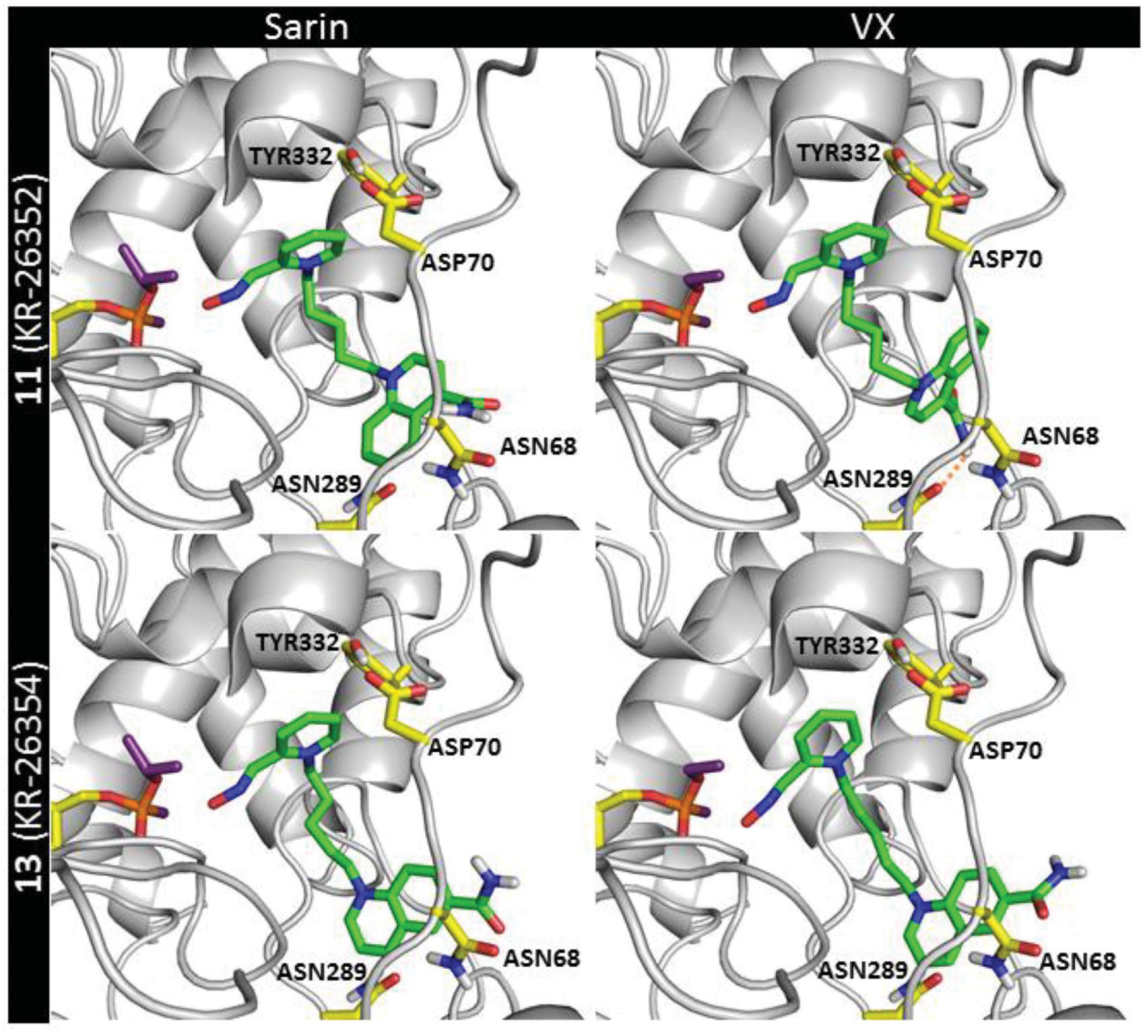
The pre-reactivation complex of compounds **11** and **13** within the *h*BChE active site blocked by sarin (NIMP) and VX (NEMP).

Further, the poses for compounds **11** and **13** within the BChE active site inhibited by sarin and VX were obtained (Figure 9). Notably, the recurring interactions to cation–π and π–π stacking with TYR332 were found decreased for both molecules. Plus further loss of binding to ASP70 in a pre-reactivation state might be the reason for the decreased reactivation ability of *hr*BChE by these compounds compared to obidoxime (Table 1). A molecular fragment that differed these two oximes from each other was the hydrogen bond formed by the amide group. This bond with ASN68 for sarin-BChE and ASN289 for VX-BChE adduct was more common in the docking poses of compound **11**. This is in line with the differences in the reactivation ability *in vitro* which was found slightly better for oxime **11**.

To sum up molecular modelling studies, they enabled to visualise the significant differences between two states occurring during enzymatic reactivation. The tested oximes were presented preferably in the conformations that did not lead to OP reactivation. On this basis, it can be concluded that shifting the balance towards the creation of the pre-reactivation complex is essential for effective reactivation of both AChE and BChE. Significantly lower values of the scoring function during the evaluation of BChE pre-reactivation complexes with sarin and VX (compared to AChE) emphasised the great difficulty to effectively reactivate this enzyme. Several key amino acid residues repeatedly participated in the formation of the pre-reactivation complex with oximes. Among them, TYR332 and ASP70 in BChE or with TYR341, ASP74, and TRP286 in AChE were highlighted. The significant impact of some of these amino acids in the reactivation process was also confirmed in mutagenesis studies^35^^,^^36^. For this reason, the shift of balance in favour of pre-reactivation complex and consequent reactivation should be done via increased interactions with these particular amino acids. The introduction of the quinolinium fragment improved interaction with the broadened entry into the BChE cavity but this fragment significantly increased the potential for new compounds to act as cholinesterase inhibitors as well. It seems that further modifications in the length of the linker between the pyridinium and quinolinium part and the introduction of substituents (that could reduce the quinolinium preference for interaction with TRP86 in AChE or TRP82 in BChE) can both influence unwanted enzyme inhibition and intensify pre-reactivation formation.

## 4. Conclusions

The pyridinium-2-carbaldoximes with quinolinium carboxamide moiety were designed and successfully prepared as the analogues of formerly studied AChE reactivators (i.e. asoxime, K117, and K127). The novel compounds showed intermediate-to-high inhibition ability of both cholinesterases with compound **11** being the less potent inhibitor. Their reactivation ability was evaluated *in vitro* on *hr*AChE and *hr*BChE inhibited by nerve agent surrogates or pesticide metabolite paraoxon. In the reactivation screening, compound **11** was able to simultaneously reactivate *hr*AChE inhibited by all used OPs and two novel compounds (**11** and **13**) were able to reactivate *hr*BChE inhibited by NIMP or NEMP. The determination of reactivation kinetics disclosed compound **11** that was proved to be excellent reactivator of POX-inhibited *hr*AChE better to obidoxime and showed increased reactivation of *hr*BChE inhibited by NIMP or NEMP, although slightly worse to obidoxime. The molecular interactions of studied reactivators were identified by *in silico* calculations. Molecular modelling results revealed the importance of creation of the pre-reactivation complex that could lead to better reactivation of both cholinesterases together with reducing particular amino acid interactions for lower intrinsic inhibition of cholinesterases by the oxime.

## Supplementary Material

Supplemental Material
