# Supplementary material for: Pyridinium-2-carbaldoximes with quinolinium carboxamide moiety are simultaneous reactivators of acetylcholinesterase and butyrylcholinesterase inhibited by nerve agent surrogates
Source: J Enzyme Inhib Med Chem. 2021 Jan 19;36(1):437–49. doi: 10.1080/14756366.2020.1869954 (PMC7822067; doi:10.1080/14756366.2020.1869954)
Supplement: Supplemental Material [file IENZ_A_1869954_SM2804.pdf]

Supplementary information

Pyridinium-2-carbaldoximes with quinolinium carboxamide moiety are simultaneous reactivators of acetylcholinesterase and butyrylcholinesterase inhibited by nerve agent surrogates

Hyun Myung Lee[1,2⊥], Rudolf Andrys[3⊥], Jakub Jonczyk[4⊥], Kyuneun Kim[1,2], Avinash G Vishakantegowda,[1] David Malinak[3], Adam Skarka[3], Monika Schmidt[3], Michaela Vaskova[3], Kamil Latka[4], Marek Bajda[4], Young-Sik Jung[1,2]*, Barbara Malawska[4]*, Kamil Musilek[3]*

[1] Division of Bio and Drug Discovery, Korea Research Institute of Chemical Technology, Daejeon 34114, Republic of Korea

[2] Department of Medicinal and Pharmaceutical Chemistry, University of Science and Technology, Daejeon 34113, Republic of Korea

[3] University of Hradec Kralove, Faculty of Science, Department of Chemistry, Rokitanskeho 62, 500 03 Hradec Kralove, Czech Republic

[4] Department of Physicochemical Drug Analysis, Chair of Pharmaceutical Chemistry, Faculty of Pharmacy, Jagiellonian University Medical College, Medyczna 9, 30-688 Krakow, Poland

⊥ These authors contributed equally to this work.

* corresponding authors

Content

1. **^{1}H NMR spectra** 2
2. **^{13}C NMR spectra** 5
3. **MS spectra** 8
4. ***In vitro* data** 11
5. ***In silico* data** 12

1. ^{1}H NMR spectra

1.1. ^{1}H NMR spectrum of **9**

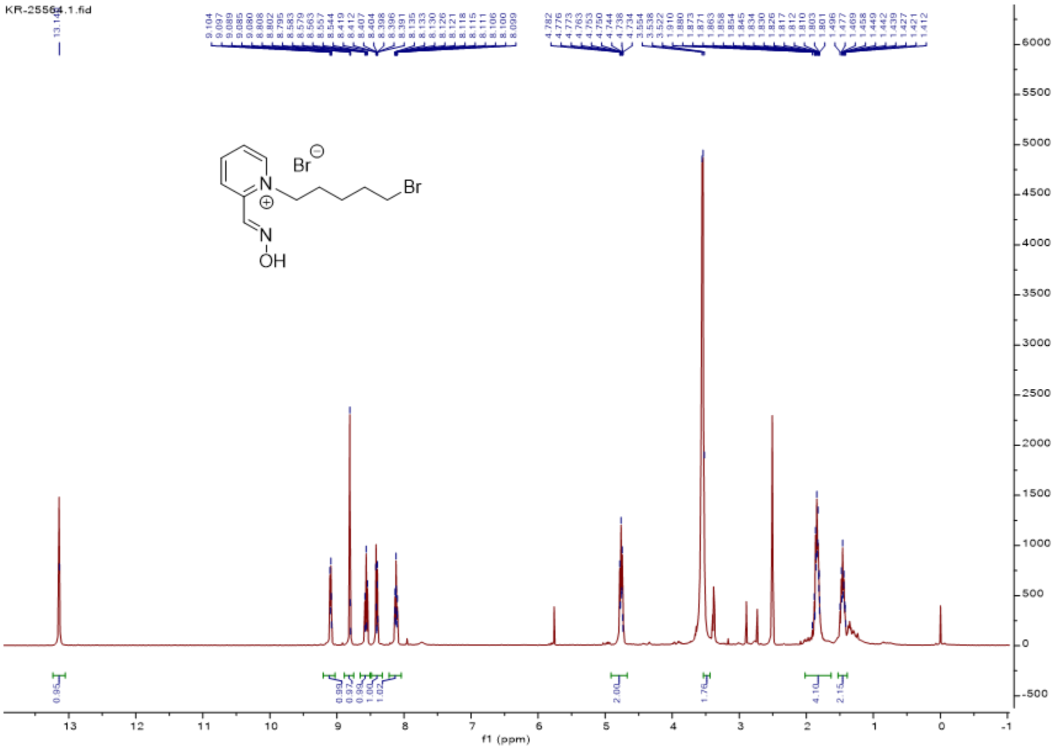

1.2. ^{1}H NMR spectrum of **10**

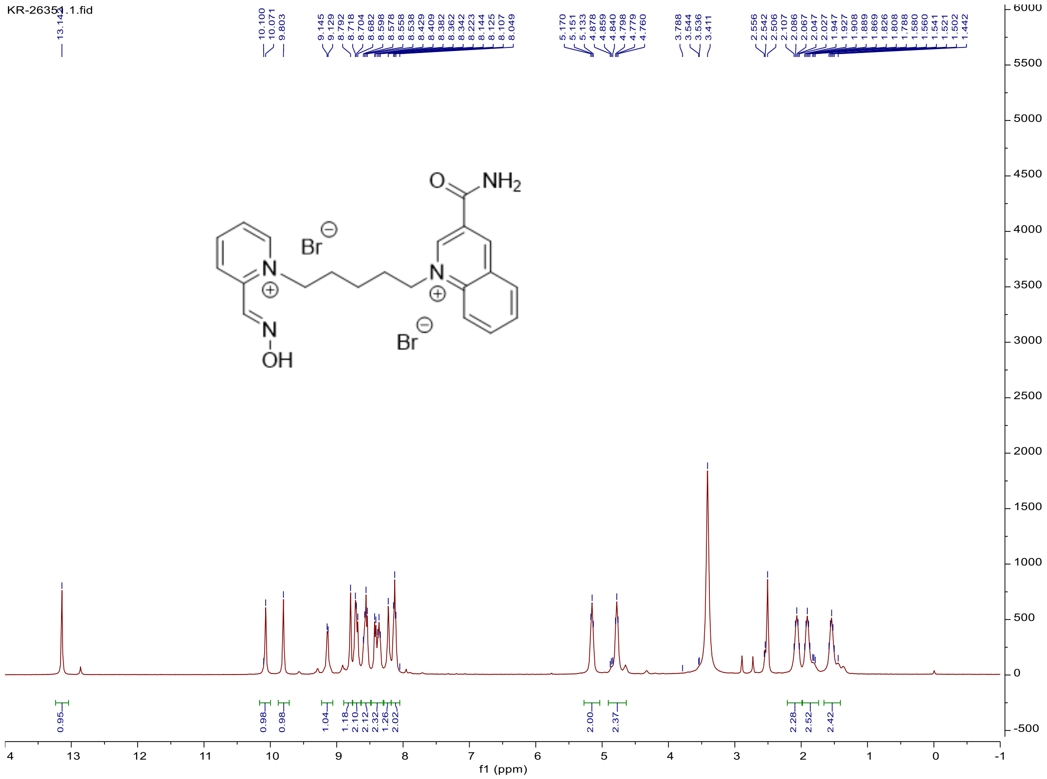

1.3. ^{1}H NMR spectrum of **11**

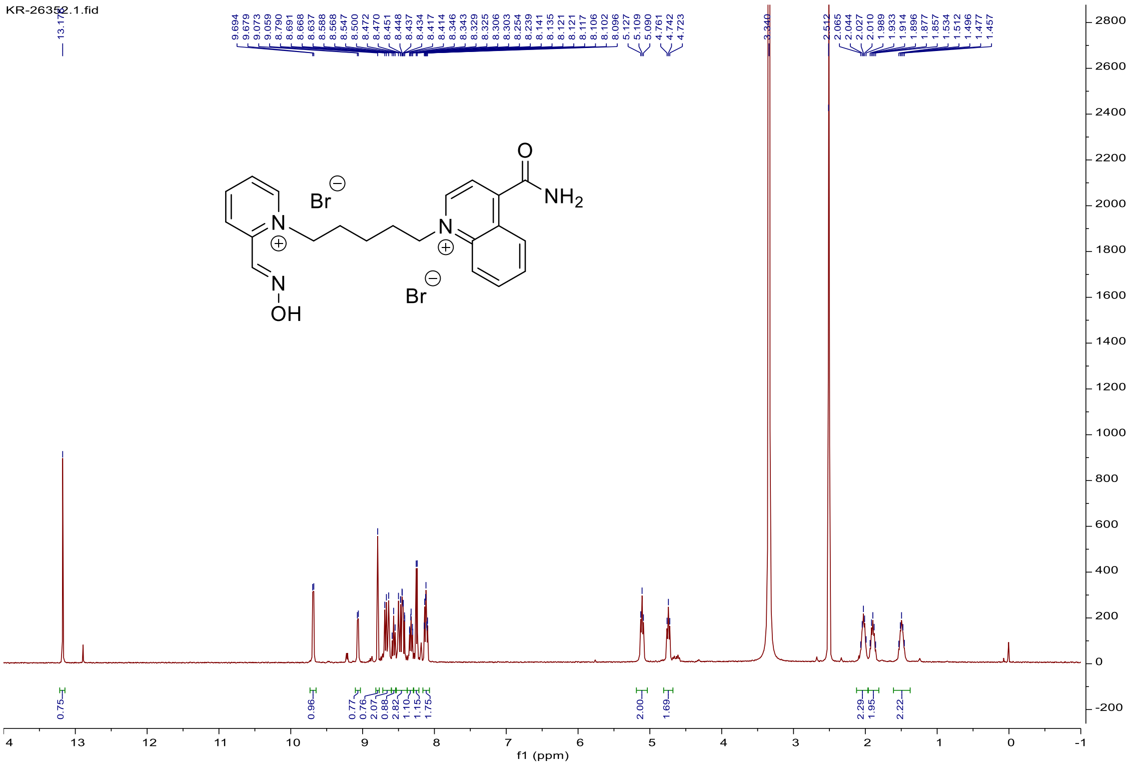

1.4. ^{1}H NMR spectrum of **12**

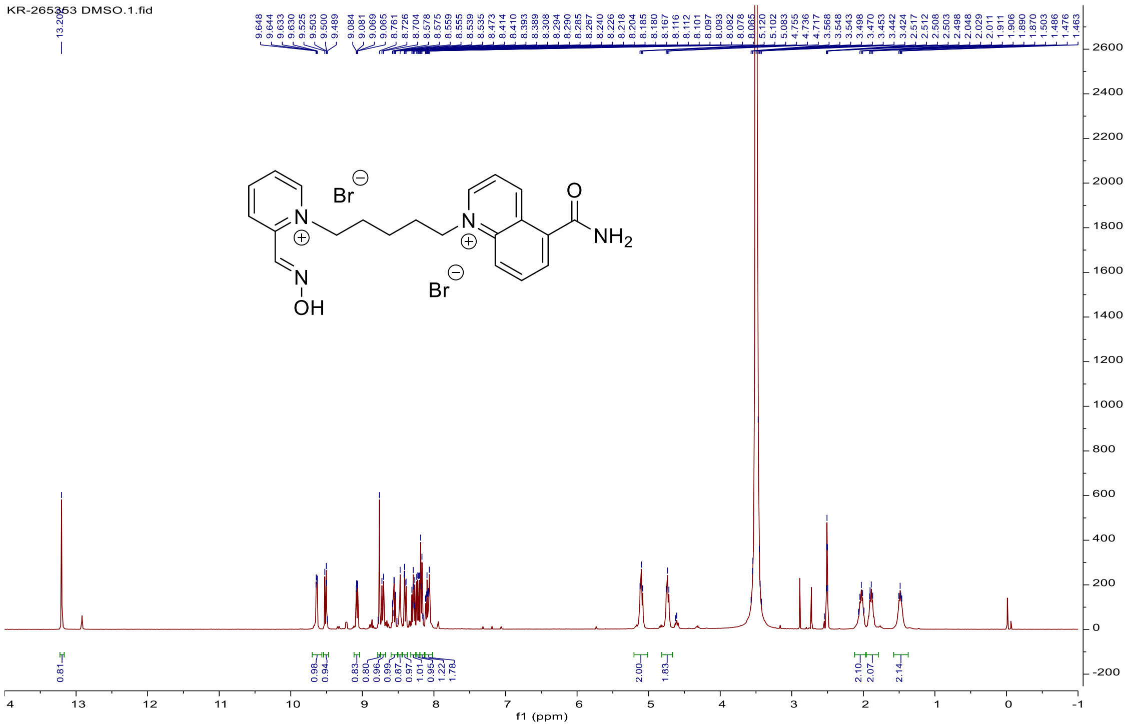

1.5. ^{1}H NMR spectrum of **13**

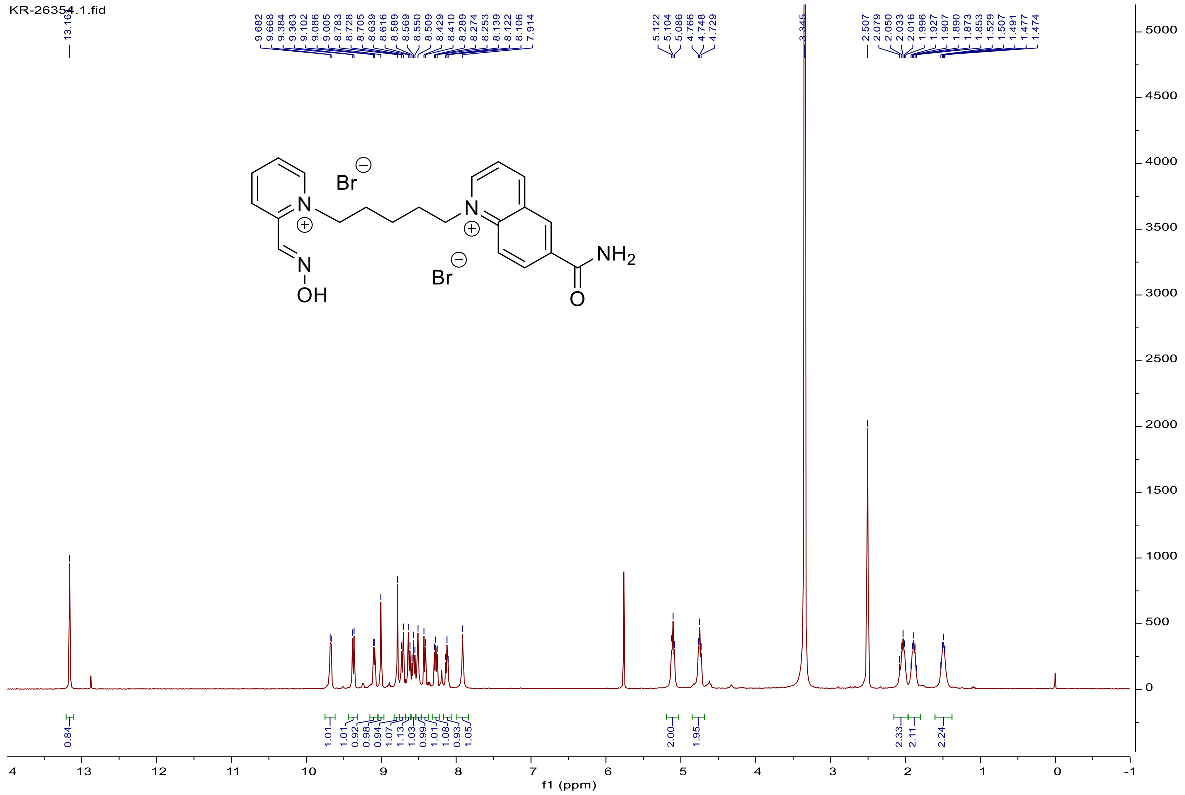

1.6. ^{1}H NMR spectrum of **14**

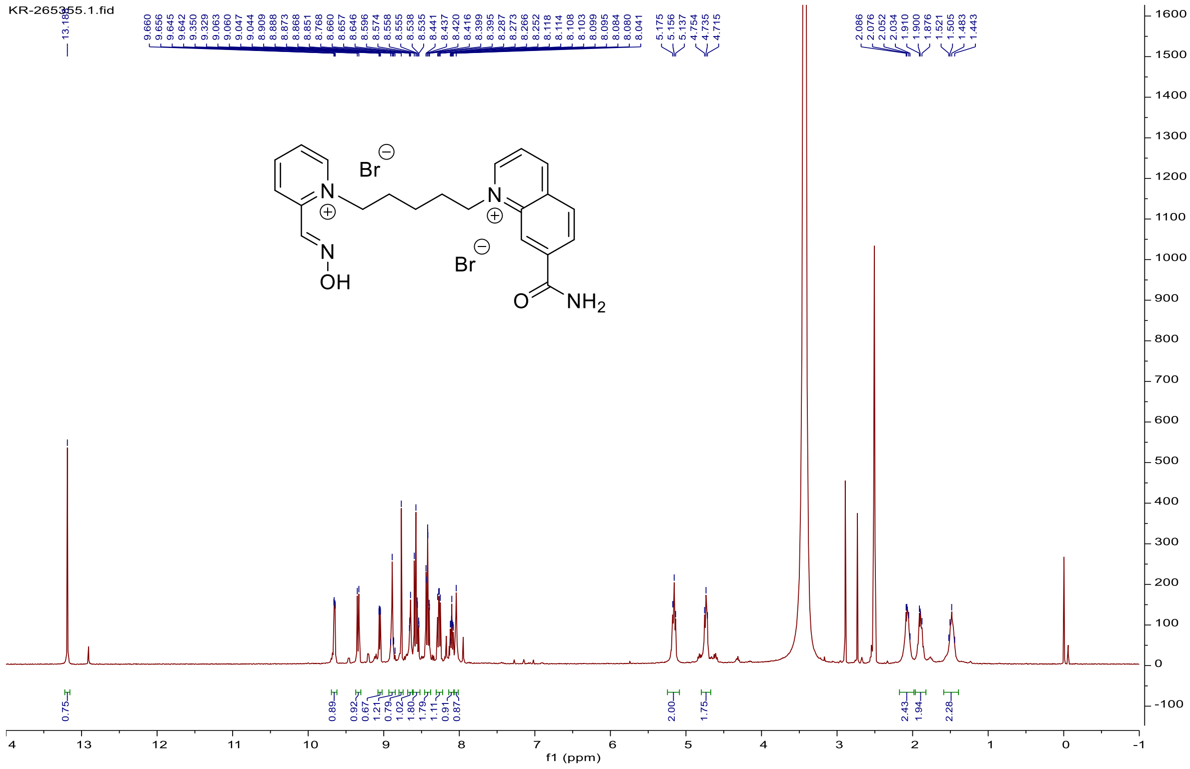

2. ^{13}C NMR spectra

2.1. ^{13}C NMR spectrum of **9**

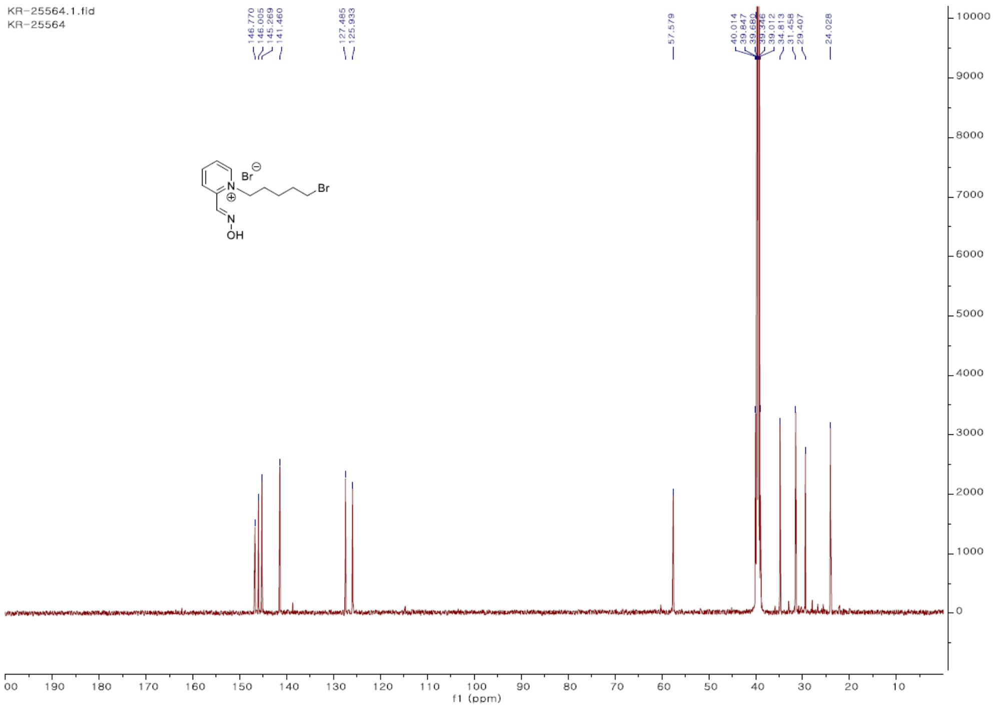

2.2. ^{13}C NMR spectrum of **10**

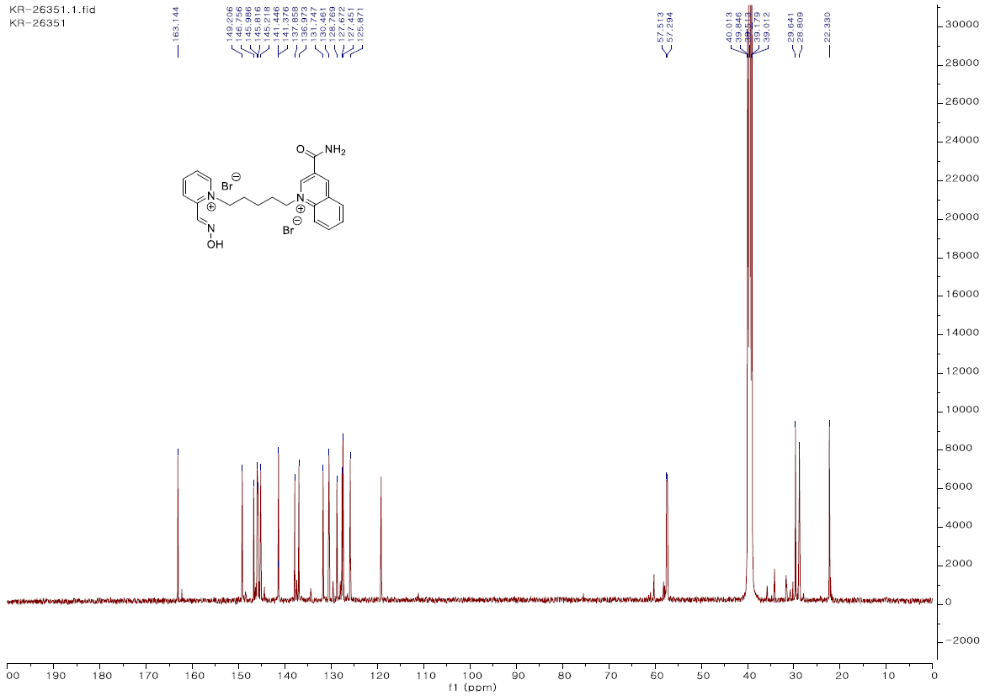

2.3. ^{13}C NMR spectrum of **11**

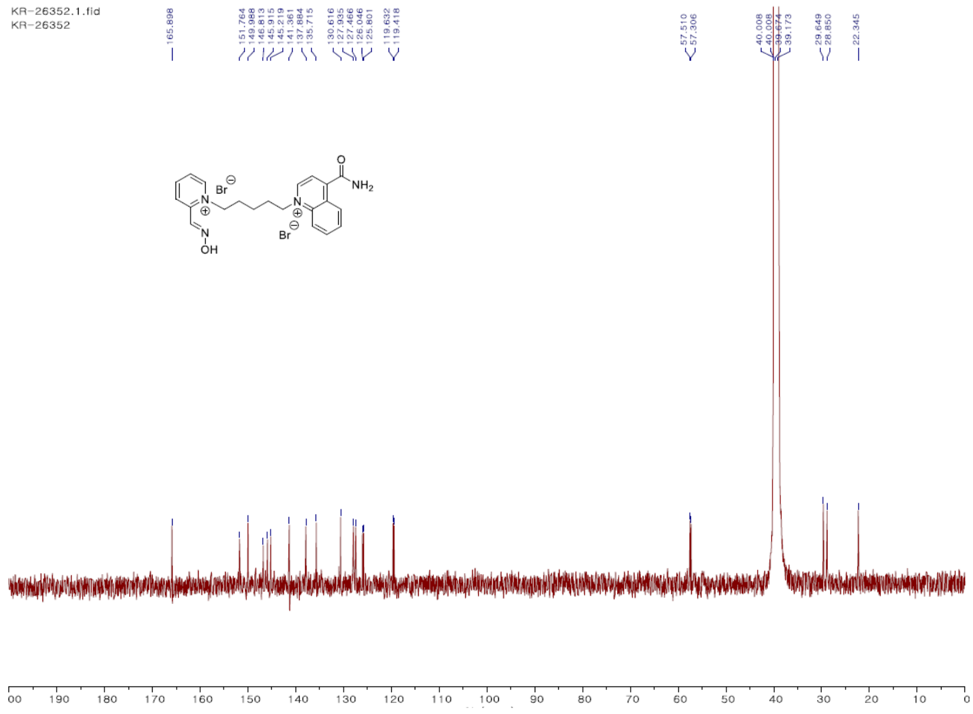

2.4. ^{13}C NMR spectrum of **12**

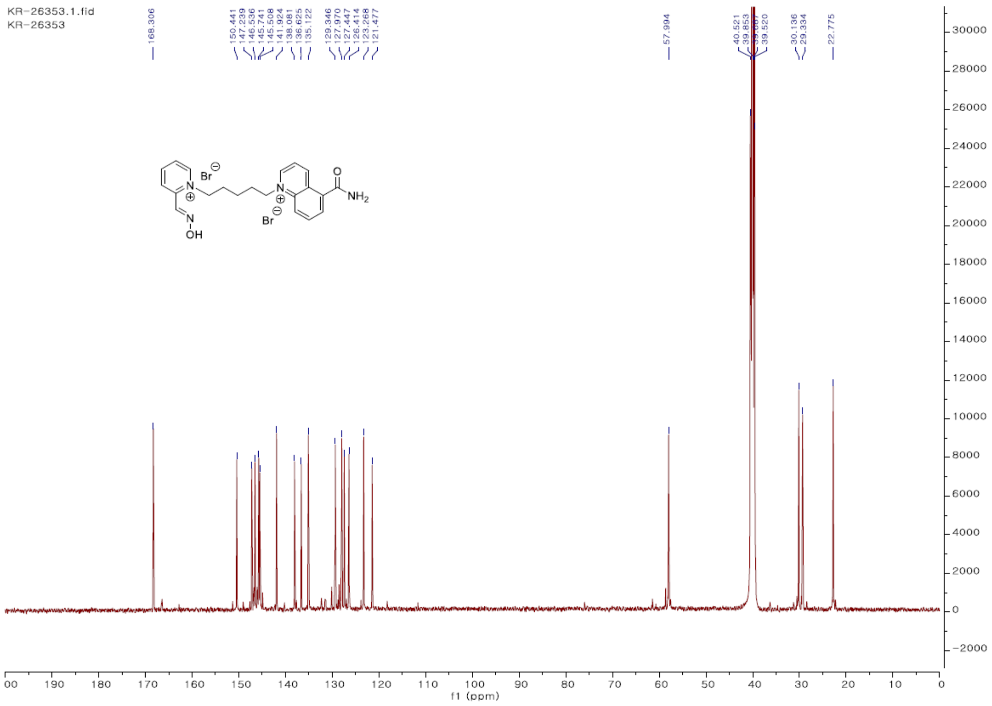

2.5. ^{13}C NMR spectrum of **13**

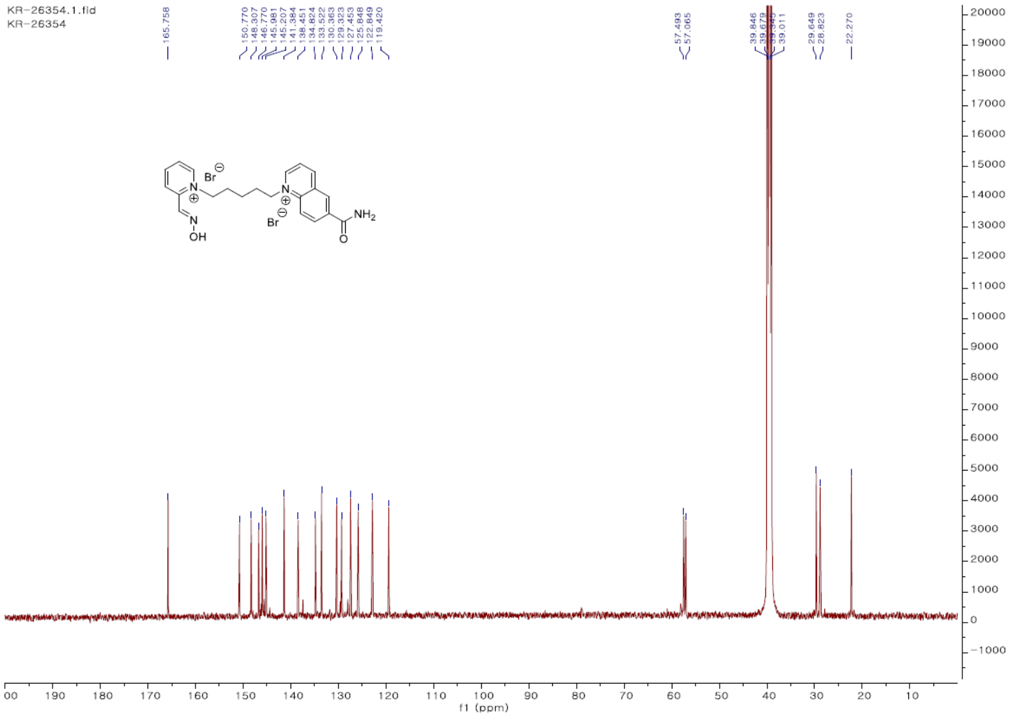

2.6. ^{13}C NMR spectrum of **14**

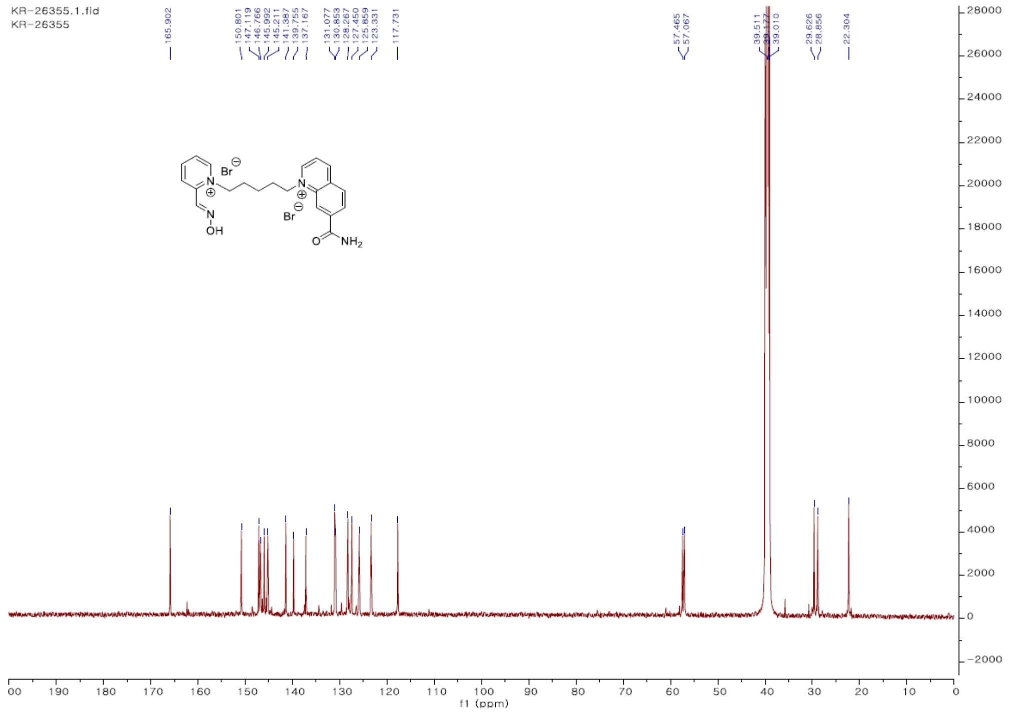

3. MS spectra

3.1. MS spectrum of **9**

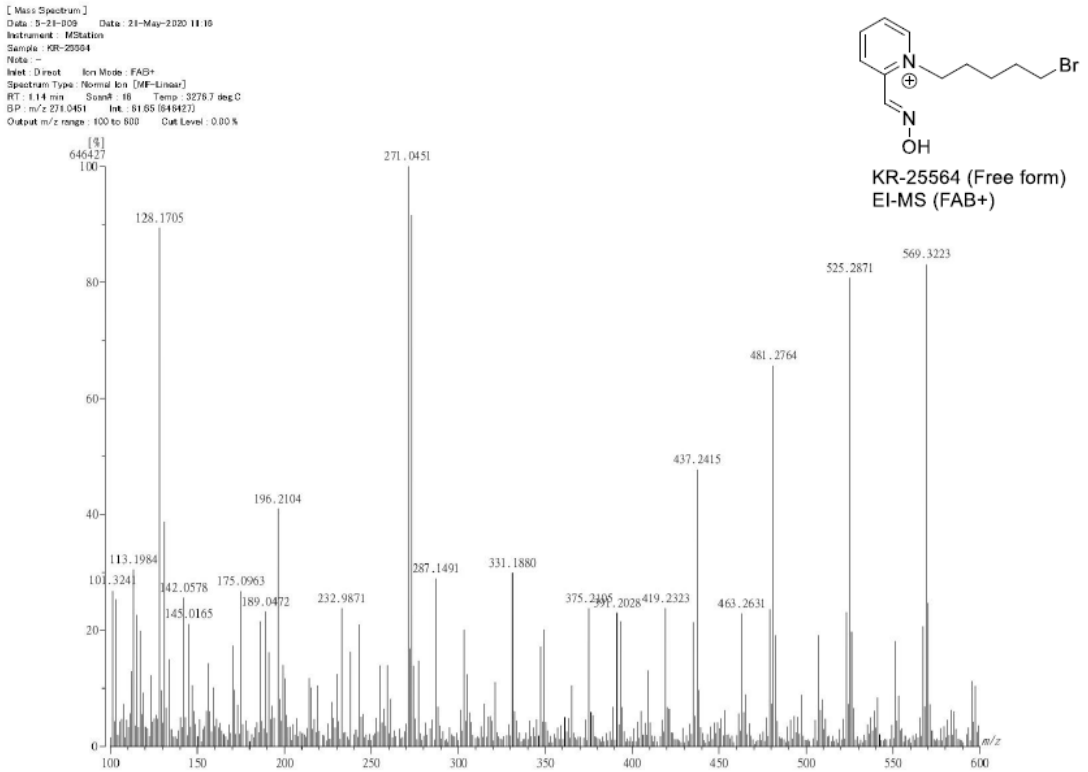

3.2. MS spectrum of **10**

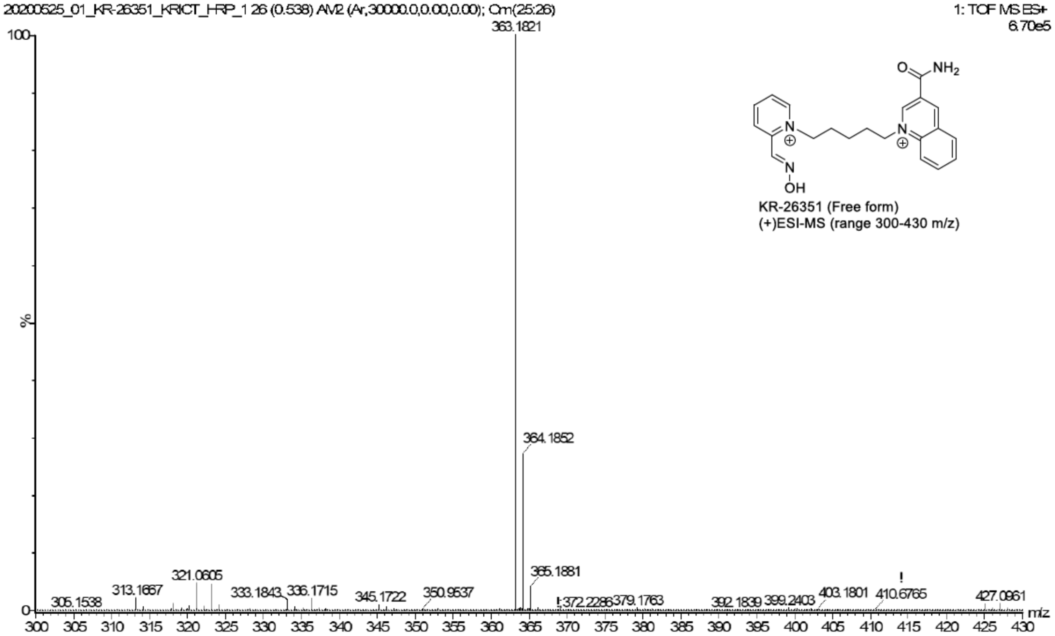

3.3. MS spectrum of **11**

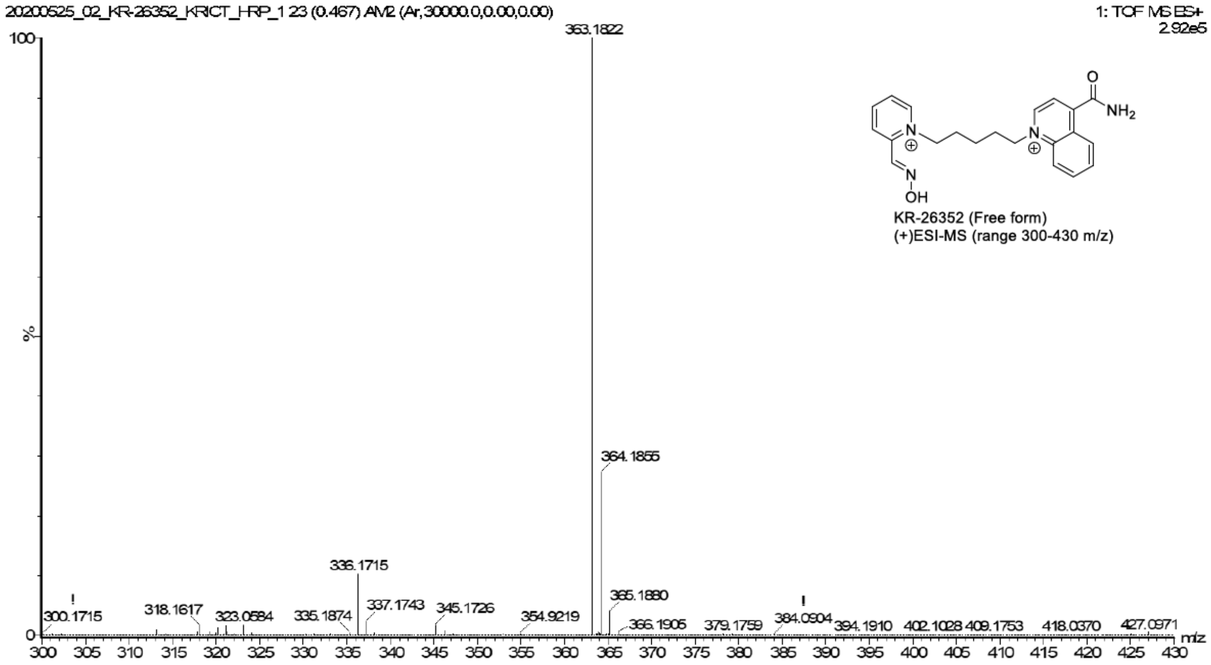

3.4. MS spectrum of **12**

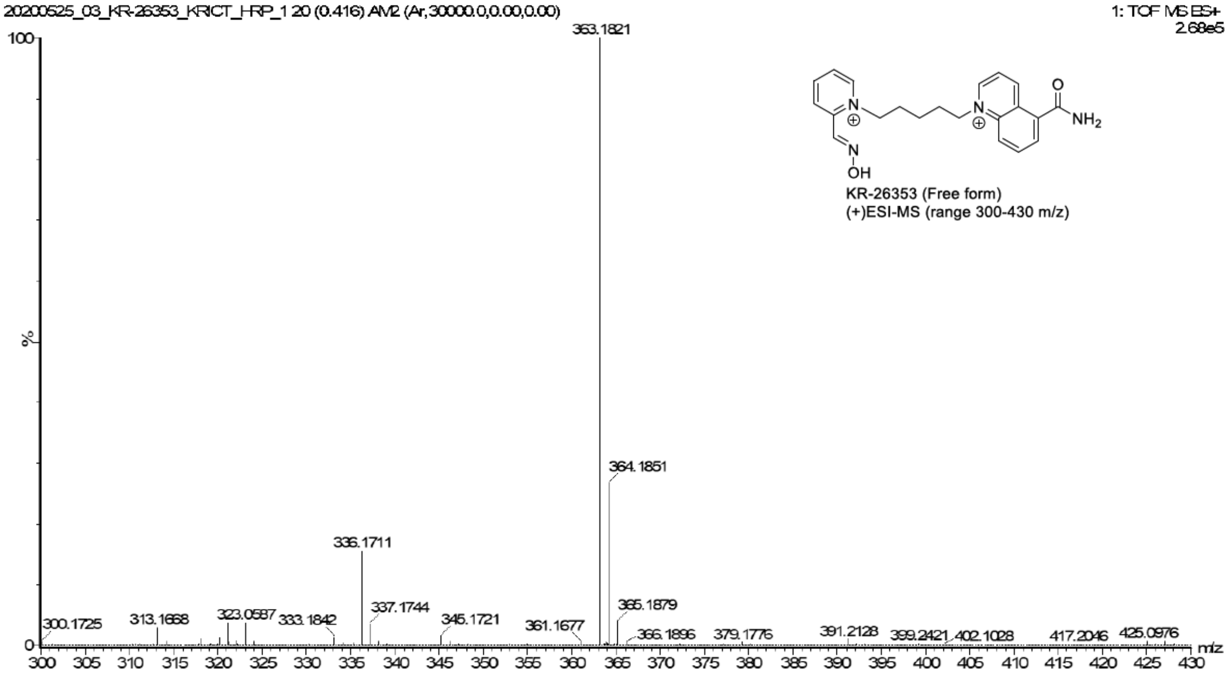

3.5. MS spectrum of **13**

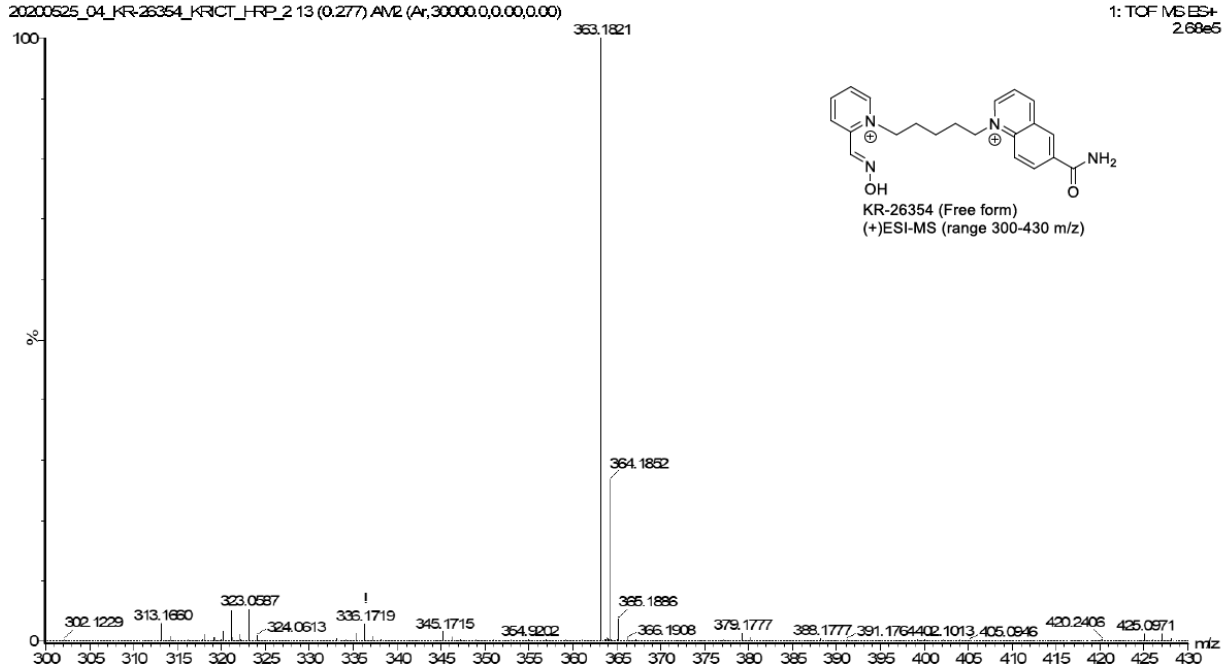

3.6. MS spectrum of **14**

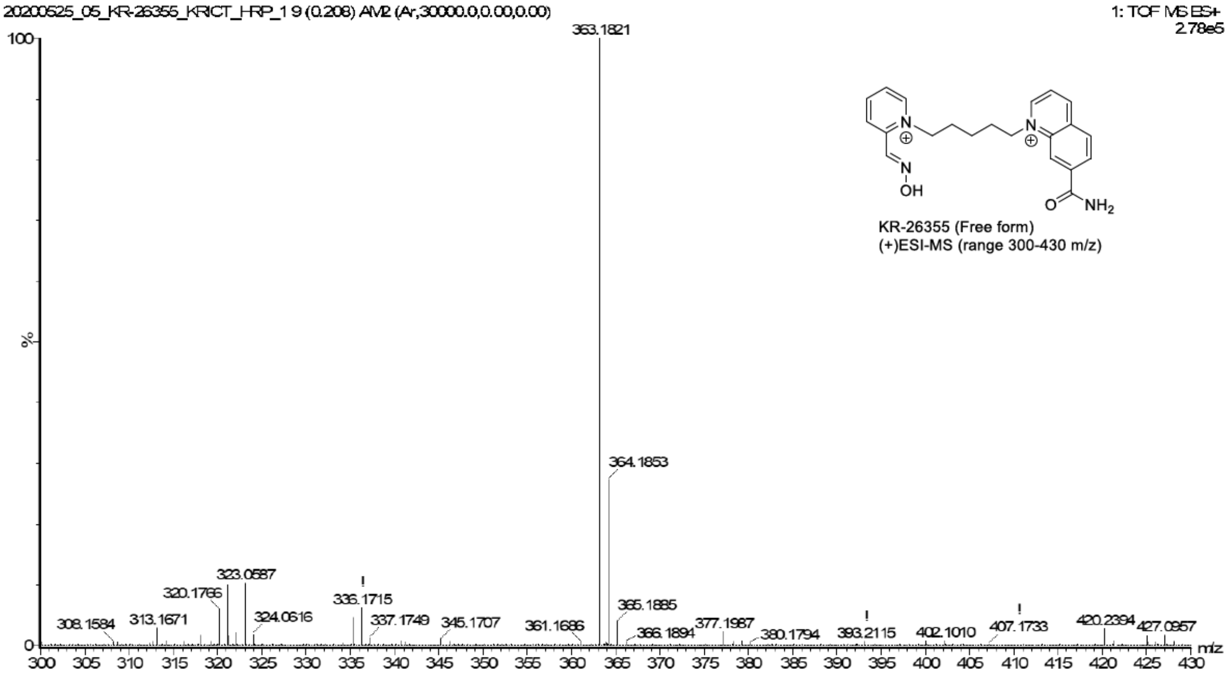

4. *In vitro* data

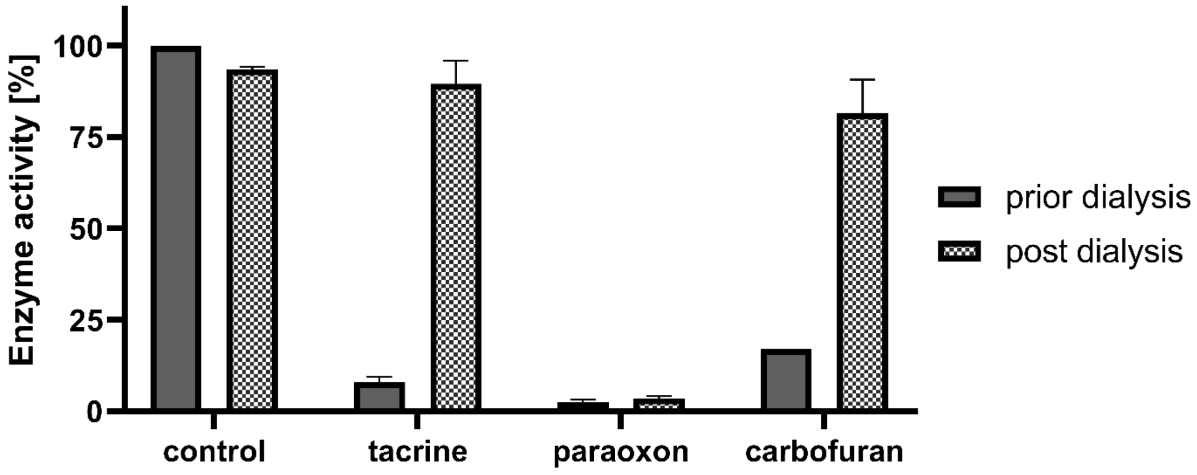

Figure 1S. Enzymatic activity of *hr*AChE prior or post dialysis – control (uninhibited) *hr*AChE, tacrine inhibited *hr*AChE (reversible competitive inhibition), paraoxon inhibited *hr*AChE (irreversible inhibition) and carbofuran inhibited *hr*AChE (reversible competitive inhibition).

5. *In silico* data

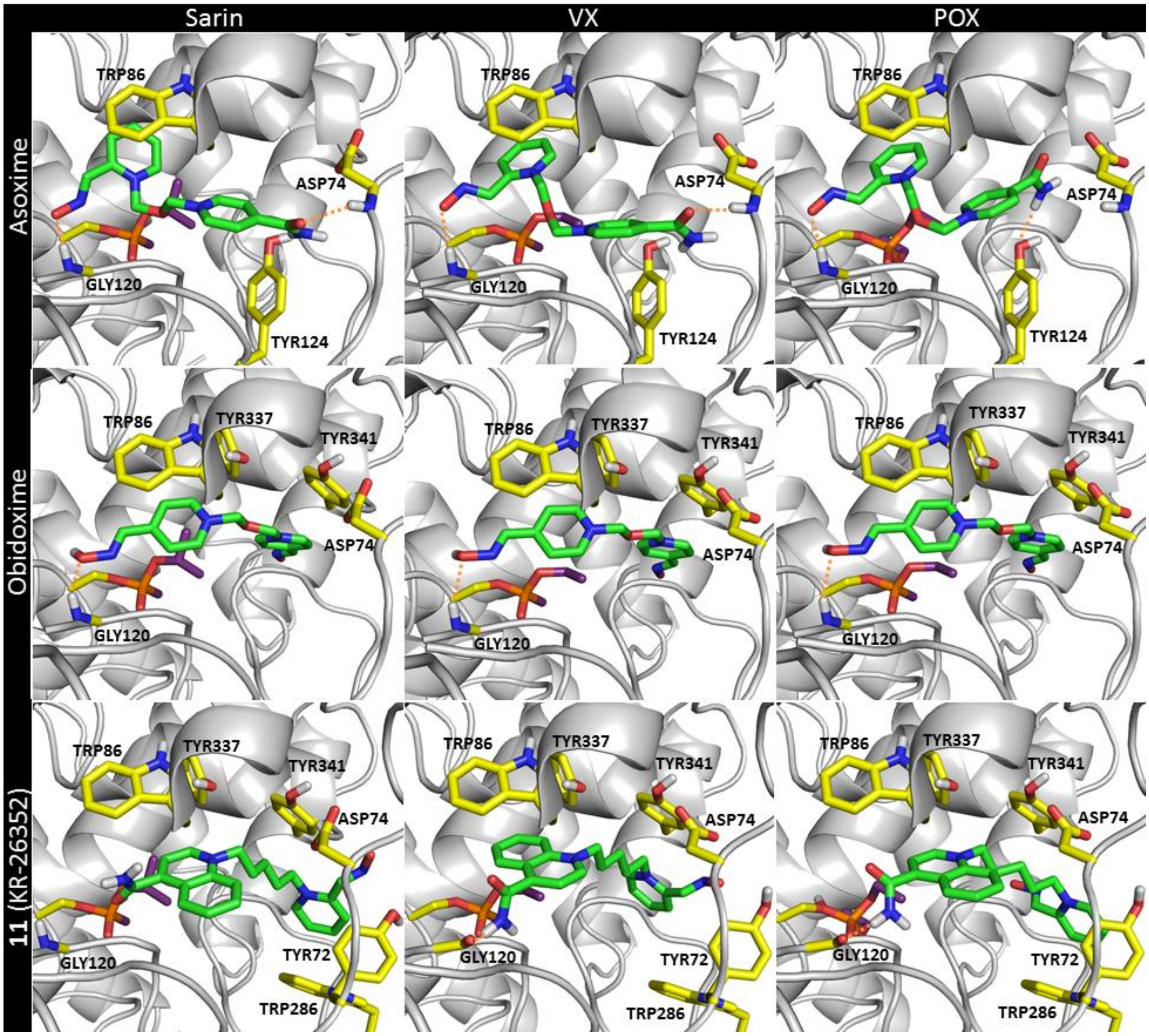

Figure 2S. Non-reactive poses of asoxime, obidoxime, and compound **11** obtained during docking to AChE inhibited by sarin (NIMP), VX (NEMP), and POX complexes.

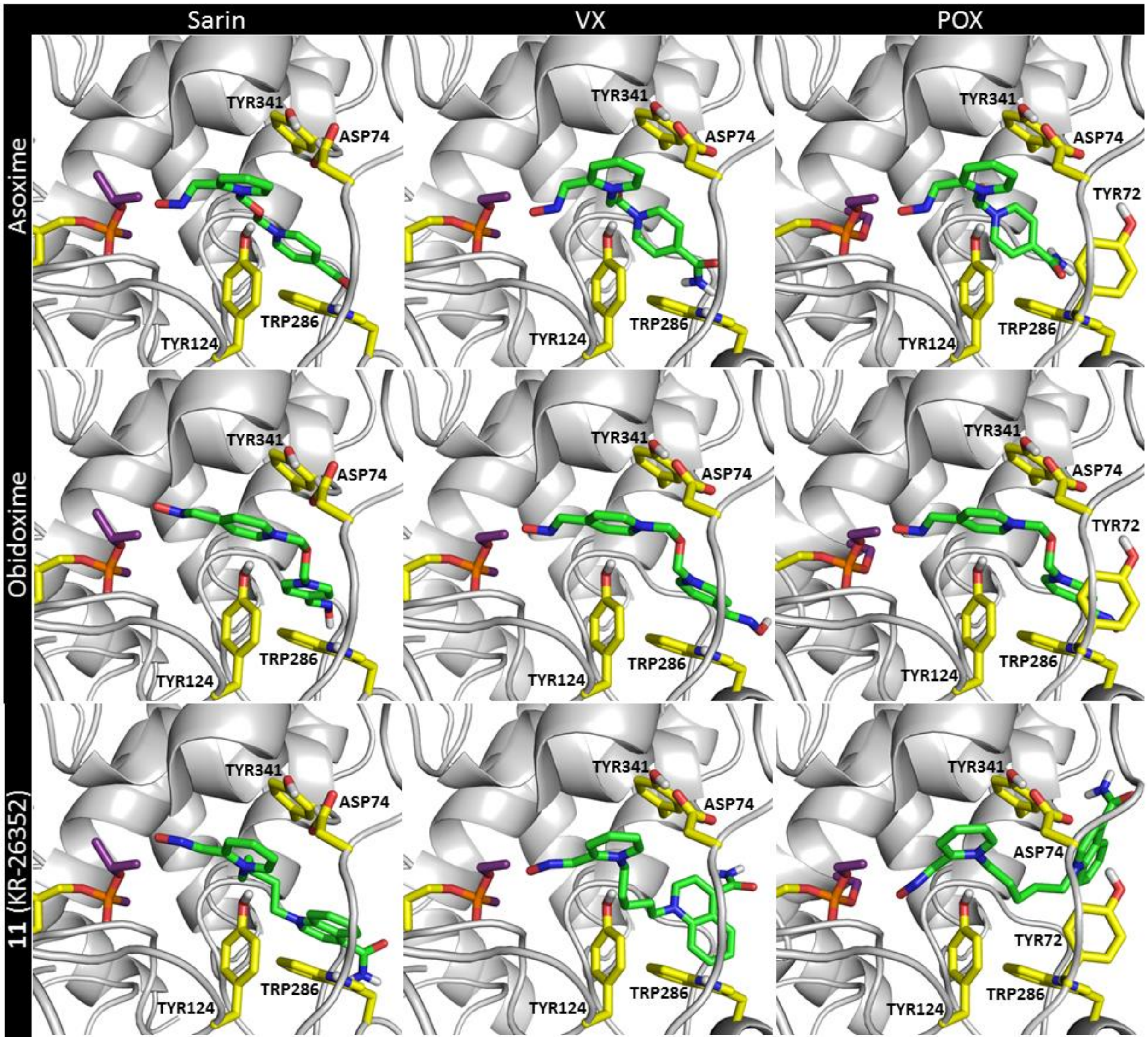

Figure 3S. Pre-reactivation poses of asoxime, obidoxime, and compound **11** in AChE blocked by sarin (NIMP), VX (NEMP), and POX.

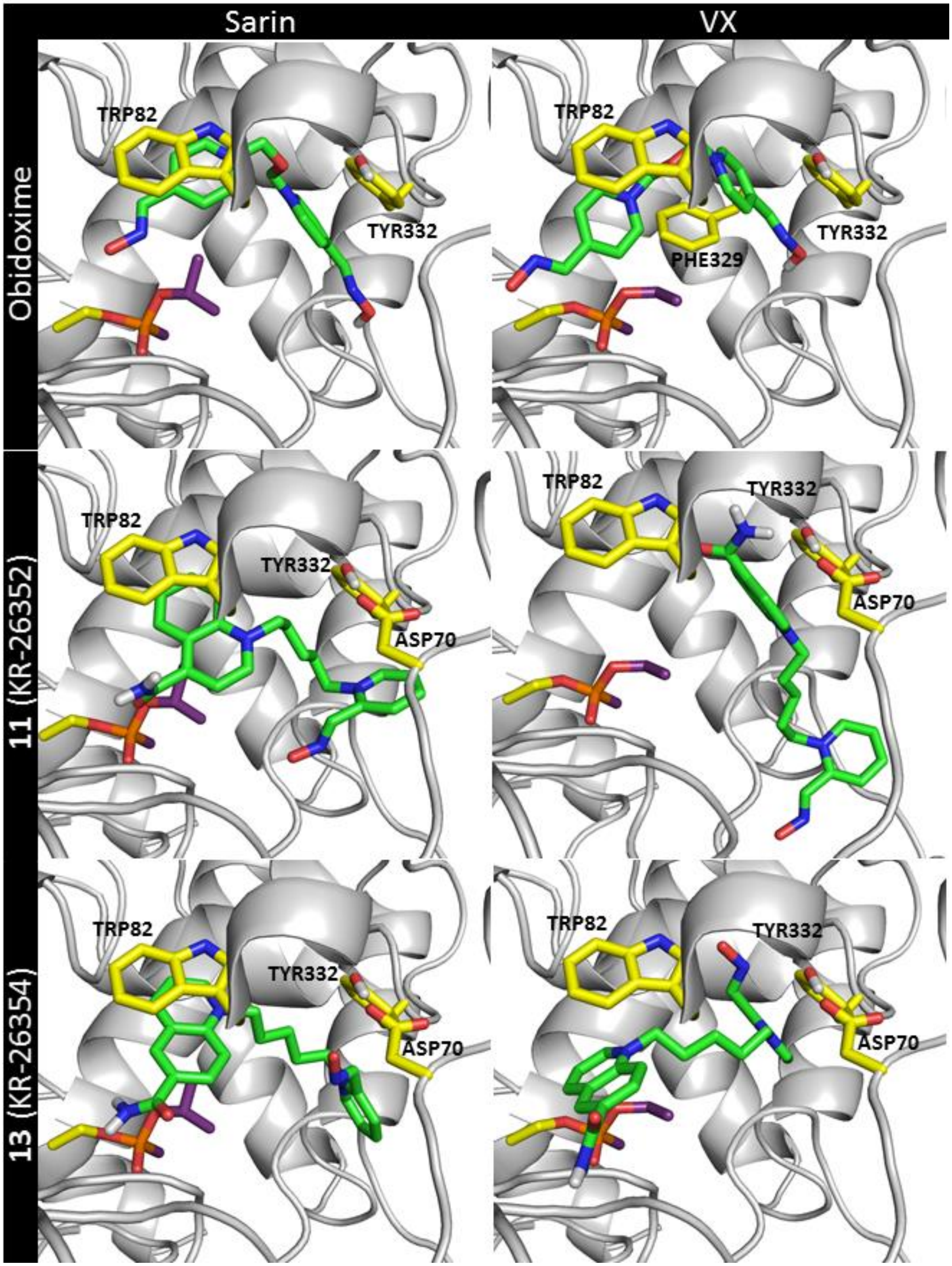

Figure 4S. Non-reactive binding mode of obidoxime, **11**, and **13** at the BChE active site blocked by sarin (NIMP) and VX (NEMP).

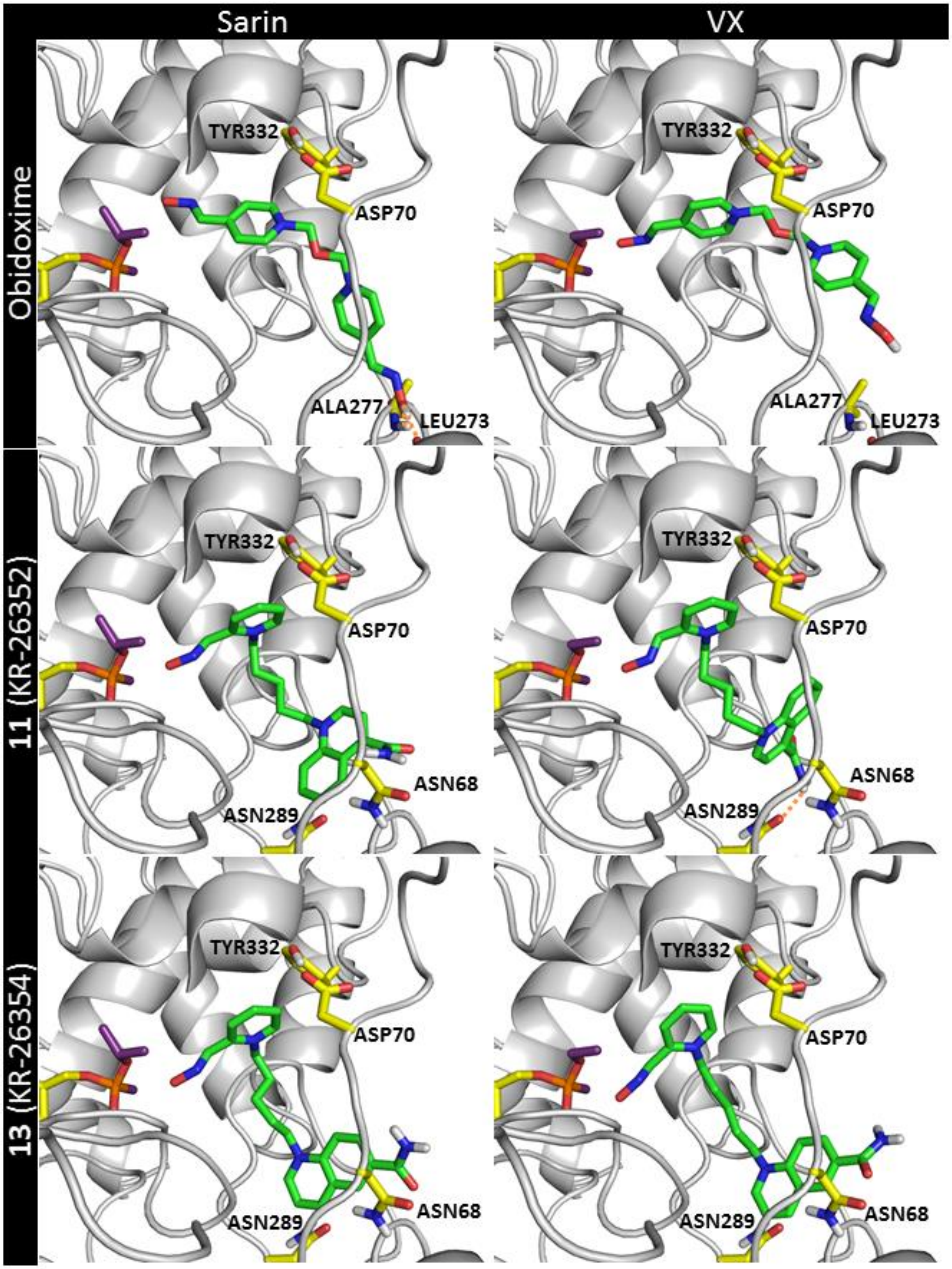

Figure 5S. Pre-reactivation complex of obidoxime, **11** and **13** within the BChE active site blocked by sarin (NIMP) and VX (NEMP).
